# Hypoxic Exosomal circPLEKHM1‐Mediated Crosstalk between Tumor Cells and Macrophages Drives Lung Cancer Metastasis

**DOI:** 10.1002/advs.202309857

**Published:** 2024-03-21

**Authors:** Dongliang Wang, Shuoer Wang, Mingming Jin, Yan Zuo, Jianpeng Wang, Ya Niu, Qian Zhou, Jiwei Chen, Xinru Tang, Wenxuan Tang, Xiyu Liu, Hang Yu, Wangjun Yan, Huan‐Huan Wei, Gang Huang, Shaoli Song, Shuang Tang

**Affiliations:** ^1^ Department of Nuclear Medicine Cancer Institute Fudan University Shanghai Cancer Center Shanghai 200032 P. R. China; ^2^ Department of Oncology Shanghai Medical College Fudan University Shanghai 200032 P. R. China; ^3^ Shanghai Engineering Research Center of Molecular Imaging Probes Shanghai 200032 P. R. China; ^4^ Center for Biomedical Imaging Fudan University Shanghai 200032 P. R. China; ^5^ Department of Musculoskeletal Oncology Fudan University Shanghai Cancer Center Shanghai 200032 P. R. China; ^6^ Shanghai Key Laboratory of Molecular Imaging Shanghai University of Medicine and Health Sciences Shanghai 201318 P. R. China; ^7^ Jiading District Central Hospital Affiliated Shanghai University of Medicine and Health Sciences Shanghai 201318 P. R. China; ^8^ Department of Critical Care Medicine The First Affiliated Hospital of Harbin Medical University Harbin 150007 P. R. China; ^9^ School of Clinical Medicine Shanghai Medical College Fudan University Shanghai 200032 P. R. China; ^10^ Bio‐med Big Data Center Chinese Academy of Sciences Key Laboratory of Computational Biology CAS Center for Excellence in Molecular Cell Science Shanghai Institute of Nutrition and Health Shanghai 200031 P. R. China; ^11^ Shanghai Key Laboratory of Radiation Oncology Shanghai 200032 P. R. China

**Keywords:** circPLEKHM1, exosomal circRNA, hypoxia, macrophage polarization, metastasis, non‐small cell lung cancers

## Abstract

Intercellular communication often relies on exosomes as messengers and is critical for cancer metastasis in hypoxic tumor microenvironment. Some circular RNAs (circRNAs) are enriched in cancer cell‐derived exosomes, but little is known about their ability to regulate intercellular communication and cancer metastasis. Here, by systematically analyzing exosomes secreted by non‐small cell lung cancer (NSCLC) cells, a hypoxia‐induced exosomal circPLEKHM1 is identified that drives NSCLC metastasis through polarizing macrophages toward to M2 type. Mechanistically, exosomal circPLEKHM1 promoted PABPC1‐eIF4G interaction to facilitate the translation of the oncostatin M receptor (OSMR), thereby promoting macrophage polarization for cancer metastasis. Importantly, circPLEKHM1‐targeted therapy significantly reduces NSCLC metastasis in vivo. circPLEKHM1 serves as a prognostic biomarker for metastasis and poor survival in NSCLC patients. This study unveils a new circRNA‐mediated mechanism underlying how cancer cells crosstalk with macrophages within the hypoxic tumor microenvironment to promote metastasis, highlighting the importance of exosomal circPLEKHM1 as a prognostic biomarker and therapeutic target for lung cancer metastasis.

## Graphical abstract

A hypoxia‐induced exosomal circRNA, circPLEKHM1, drives lung cancer metastasis by educating macrophages in the tumor microenvironment. circPLEKHM1 functions as a scaffold for PABPC1‐eIF4G interaction to facilitate the translation of the oncostatin M receptor, polarizing macrophages towards to M2 type for cancer metastasis. circPLEKHM1 is a significant prognostic biomarker and effective therapeutic target for M2 macrophage‐involved cancer metastasis.

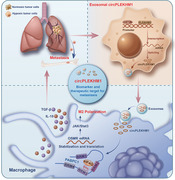

## 1 Introduction

Metastasis is the principal cause of cancer death and involves critical interactions between tumor cells and the tumor microenvironment (TME).^[^ ^1^, ^2^, ^3^, ^4^ ^]^ Lung cancer is the leading cause of cancer‐related deaths worldwide.^[^ ^5^ ^]^ Bone metastases occur in ≈50% of advanced‐stage NSCLC patients, with very limited treatment options.^[^ ^6^ ^]^ Treatment of metastasis remains a major clinical challenge.

Hypoxia and tumor‐infiltrating immune cells are potent microenvironmental factors that promote metastasis.^[^ ^1^, ^7^ ^]^ As the predominant immune cells in the TME, macrophages preferentially cluster in the hypoxic regions of the tumor, where they polarize into M2‐type tumor‐associated macrophages,^[^ ^8^ ^]^ and communicate with tumor cells to promote cancer progression and metastasis.^[^ ^3^, ^9^, ^10^ ^]^ However, the mechanism through which macrophages are instructed by the hypoxic TME to regulate cancer metastasis remains largely unknown. Additionally, approaches that block tumor cell‐macrophage communication to fight cancer metastasis are lacking.

Tumor‐secreted exosomes are small extracellular vesicles (30–150 nm in size) that act as critical mediators of intercellular communication between tumor cells and TME. They often regulate cancer metastasis by delivering proteins, DNA, or non‐coding RNA to adjacent cells or tissues.^[^ ^11^, ^12^, ^13^ ^]^ Emerging evidence has shown that tumor exosomes are enriched with circular RNAs (circRNAs),^[^ ^14^, ^15^ ^]^ raising the interesting hypothesis that exosomal circRNAs may serve as molecular messengers that mediate intercellular communication in the TME. CircRNAs are mainly produced by pre‐mRNA back‐splicing and are generally more stable than linear mRNAs.^[^ ^16^, ^17^ ^]^ There is a growing interest in circRNAs because of their unique structure and conformation, directing further research to explore their potential as therapeutic agents and biomarkers.^[^ ^18^, ^19^ ^]^ Notably, circRNAs play pivotal roles in the development of cancer through different functional mechanisms, including acting as miRNA sponges, forming functional complexes, interacting with RNA‐binding protein (RBP), and coding proteins.^[^ ^20^, ^21^ ^]^ For example, the depletion of circRNA CDR1as was identified to promote melanoma metastasis as an interactor and regulator of the RNA binding protein IGF2BP3.^[^ ^22^ ^]^ However, the exact role of most exosomal circRNAs in cancer metastasis remains unknown. In particular, whether and how hypoxia‐responsive exosomal circRNAs regulate the crosstalk between tumor and immune cells within the TME to mediate cancer metastasis remains elusive.

Herein, we systematically analyzed exosomes derived from NSCLC under hypoxic conditions and identified exosomal circPLEKHM1 as a hypoxia‐induced circRNA that plays a vital role in promoting metastasis by inducing M2 macrophage polarization. NSCLC‐secreted exosomal circPLEKHM1 was activated by HIF1A to promote OSMR translation and the JAK/STAT3 pathway by enhancing the PABPC1‐eIF4G interaction, thereby polarizing macrophages to the M2‐type within the TME. Notably, we developed a new ^125^I‐CD115 radioactive probe for micro‐SPECT/CT imaging, which demonstrated that circPLEKHM1 can induce M2 polarization in macrophages in vivo. Strikingly, CircPLEKHM1‐based therapy dramatically reduced NSCLC metastasis through macrophages in vivo. CircPLEKHM1 served as a strong biomarker of metastasis and poor prognosis in NSCLC patients. This study uncovered a new exosomal circRNA‐mediated mechanism that regulates cancer‐macrophage communication in the hypoxic TME to promote NSCLC cancer metastasis. We highlighted the potential application potential of circPLEKHM1 not only as a prognostic biomarker but also as an effective therapeutic target for macrophage‐involved lung cancer metastasis.

## 2 Results

### 2.1 CircRNA Profiling Reveals circPLEKHM1 is Enriched in NSCLC‐Derived Exosomes under Hypoxia

**Figure 1:**
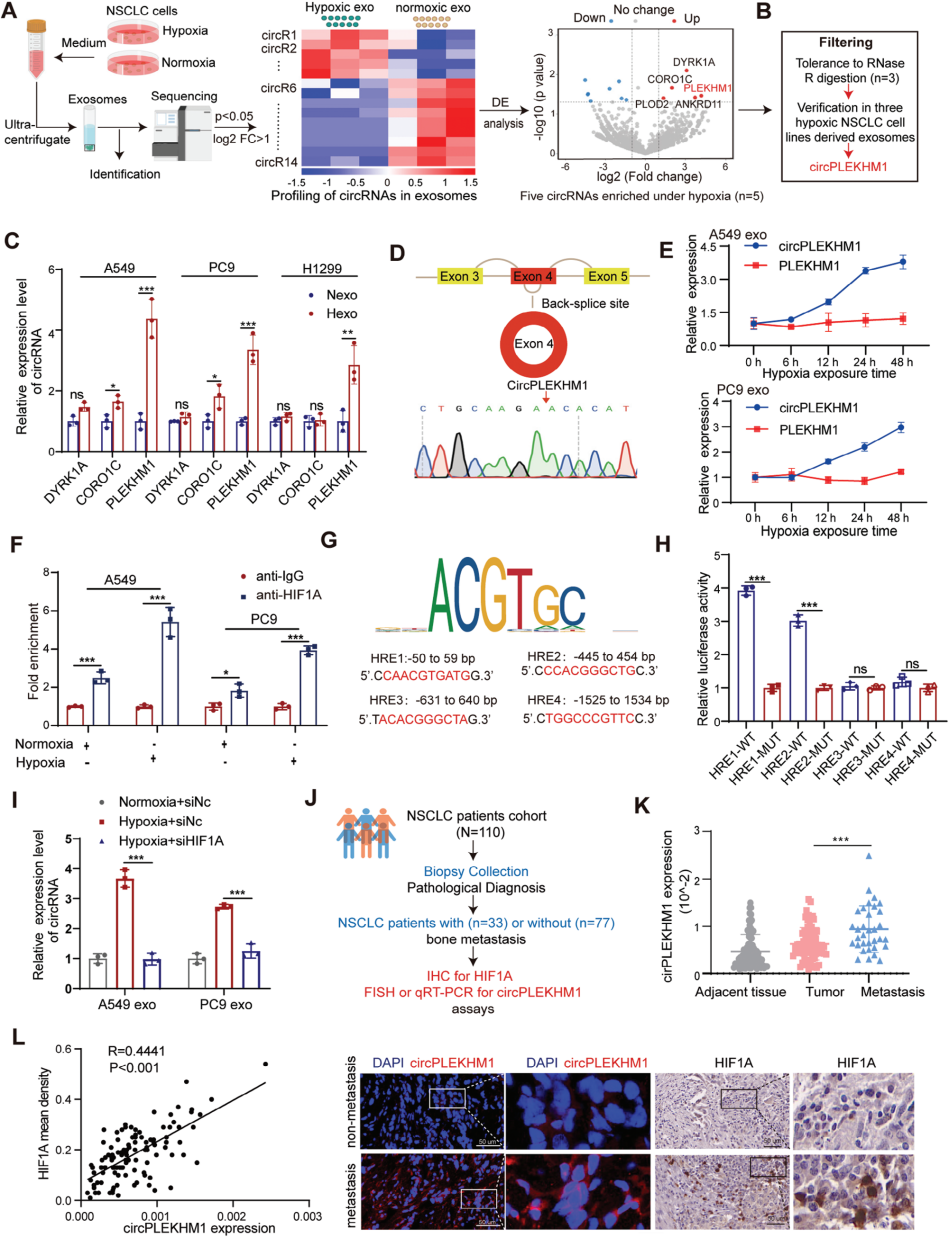
circRNA Profiling of NSCLC‐derived exosomes identifies circPLEKHM1 as a hypoxia‐induced circRNA that is upregulated by HIF1A. A) circRNA sequencing on exosomes derived from NSCLC culture medium under hypoxia or normoxia to identify hypoxia‐responsive exosomal circRNAs. Flow diagram demonstrating the study design for exosome extraction and circRNA sequencing mapping and screening (left panel). The hierarchical cluster thermogram shows the most differently expressed circRNAs between the NSCLC‐secreted hypoxic exosomes and normoxic exosomes, with the top 14 up‐regulated or down‐regulated genes selected (middle panel). Volcanic plot of circRNA expression in hypoxic‐ and normoxic‐ exosomes (log_2_(Fc) > 1.0, *p* < 0.05). The red indicates upregulation, and the blue indicates downregulation (right panel). B) Screening process to identify exosomal circPLEKHM1 as a hypoxia‐enriched circRNA candidate. C) Expression of the top three enriched exosomal circRNAs in the three lung cancer cell lines under hypoxia. D) Graphic illustration of the genome position and splicing mode for circPLEKHM1. The splicing junction site was confirmed by Sanger sequencing. E) circPLEKHM1 and PLEKHM1 expression levels in NSCLC‐derived exosomes exposed to indicated time under hypoxia. F) ChIP assays to evaluate the interactions between HIF1A and PLEKHM1 promoter region under hypoxia. G) HREs of the host gene PLEKHM1 obtained from the JASPAR database. Dual‐luciferase reporters were constructed with either of the four putative PLEKHM1 HREs or matched mutant HREs in the PLEKHM1 promoter region. H) Luciferase intensity of 293T cells co‐transfected with the indicated luciferase reporter plasmid and HIF1A plasmid. I) CircPLEKHM1 expression levels in NSCLC‐derived exosomes upon loss of HIF1A. J) Schematic diagram of the cohort establishment. K) CircPLEKHM1 expression levels in tumor tissues, matched normal tissues, and bone metastasis from NSCLC patients, measured by RT‐qPCR. L) Correlation between circPLEKHM1 and HIF1A expression in tumor tissues from NSCLC patients. Data in (C,F,H) were presented as mean ± SD, *p < 0.05, **p < 0.01, ***p < 0.001 by two‐tailed Student's *t*‐test. Data in (I,K) were presented as mean ± SD, ***p < 0.001 by one‐way ANOVA test.

Exosomes are important information carriers for cell‐to‐cell communication and can support cancer cells in adapting to hypoxia and initiating metastasis in the TME. To investigate how hypoxia regulates exosomal circRNAs in NSCLC, we performed circRNA‐seq on exosomes isolated from the culture medium of A549 cells under hypoxic (Hexo) and normoxic (Nexo) conditions (**Figure** **1**A). Transmission electron microscopy, nanoparticle tracking analysis, and western blotting confirmed that the extracted exosomes exhibited cup‐like structures, ranging from 50 to 150 nm in diameter, and expressed the common exosomal markers CD63, TSG101, and CD9 (Figure S1A–C, Supporting Information). Compared to the normoxia‐induced exosomes, hypoxia caused a significant alteration in 14 exosomal circRNAs (fold change > 2, *p <* 0.05), with five up‐regulated and nine down‐regulated (Figure 1A). Interestingly, three of the five upregulated exosomal circRNAs were resistant to ribonuclease R (RNase R) (Figure S1D, Supporting Information). Furthermore, RT‐qPCR analysis of exosomes isolated from the culture supernatants of the NSCLC cell lines A549, PC9, and H1299 showed that circPLEKHM1 was the top hypoxia‐enriched circRNA in NSCLC‐secreted exosomes (Figure 1B,C).

circPLEKHM1 is generated from exon 4 of the gene *PLEKHM1* (Pleckstrin homology domain‐containing protein family member 1). PLEKHM1 is identified as a protein adaptor for the endolysosomal system.^[^ ^23^, ^24^ ^]^ It localizes on human chromosome 17 and is 627 bp in length with a head‐to‐tail splice junction, as evidenced by Sanger sequencing (Figure 1D). To confirm the circular form of circPLEKHM1, we designed divergent primers that could amplify the head‐to‐tail splicing site and found that circPLEKHM1 was successfully amplified only from cDNA and not from genomic DNA (Figure S1E, Supporting Information). Additionally, we treated NSCLC cells with actinomycin D and observed that circPLEKHM1 was more stable than linear PLEKHM1 transcripts (Figure S1F, Supporting Information), which is in line with the notion that high stability is a feature of circRNAs. Taken together, these results suggest that circPLEKHM1 was a stably enriched circRNA in hypoxic exosomes derived from NSCLC cells.

### 2.2 Exosomal circPLEKHM1 is Upregulated by HIF1A under Hypoxia, and the circPLEKHM1‐HIF1A Axis is Highly Expressed in Bone Metastasis of NSCLC Patients

To further understand the interplay between the regulation of NSCLC‐secreted exosomal circPLEKHM1 and hypoxia in lung cancer, we investigated how HIF1A, the master transcription factor under hypoxic conditions, regulates circPLEKHM1 expression. Host linear genes of exon‐derived circRNAs were generated using the same pre‐mRNA.^[^ ^25^ ^]^ We hypothesized that HIF1A activates the transcription of the host linear gene, leading to an abundance of circPLEKHM1 in exosomes under hypoxic conditions. Indeed, the expression level of circPLEKHM1 is highly correlated with that of its host linear gene *PLEKHM1* (Figure S1G, Supporting Information). Moreover, along with the increased protein levels of HIF1A under hypoxia (Figure S1H, Supporting Information), the expression levels of circPLEKHM1 increased upon hypoxia in a time‐dependent manner in both A549 and PC9 NSCLC cells (Figure S1I, Supporting Information). Interestingly, while the expression of the host gene *PLEKHM1* in exosomes remained stably low, the levels of circPLEKHM1 in exosomes extracted from the culture medium of both A549 and PC9 NSCLC cells increased dramatically, upon hypoxia in a time‐dependent manner (Figure 1E). These results suggested that NSCLC‐secreted exosomal circPLEKHM1 was induced by hypoxia.

Next, we investigated whether HIF1A could transcriptionally regulate the *PLEKHM1* gene, the host gene of circPLEKHM1. First, the ChIP assay suggested that HIF1A could bind to the promoter region of host gene *PLEKHM1* under hypoxia in both A549 and PC9 cells (Figure 1F). A dual‐luciferase reporter assay further indicated that overexpression of HIF1A (ΔODD), a HIF1A mutant that lacks the oxygen‐degradation domain and sustains HIF1A protein stability and transaction activity, activated PLEKHM1 promoter‐driven luciferase activity under normoxia (Figure S1J, Supporting Information). These results suggested that HIF1A could bind to the *PLEKHM1* gene for transcriptional regulation. Moreover, using promoter sequence analysis tools (UCSC and JASPAR), four potential hypoxia‐responsive elements (HREs) were recognized in the promoter of *PLEKHM1* (Figure 1G). A dual‐luciferase reporter assay in 293T cells showed that the luciferase activity was enhanced in the reporters containing HRE1 or HRE2 in the presence of HIF1A overexpression or hypoxia (Figure 1H, and Figure S1K, Supporting Information), whereas reporters containing mutant HREs showed no changes despite overexpression or knockdown of HIF1A (Figure 1H, and Figure S1K,L, Supporting Information). Furthermore, the ChIP assay showed that HIF1A could bind to HRE1 and HRE2, but not to HRE3 and HRE4, in the promoter region of the host gene *PLEKHM1* (Figure S1M, Supporting Information). These results indicated that HIF1A can directly interact with HRE1 and HRE2 transcription initiation sites of *PLEKHM1*. Additionally, hypoxia‐induced intracellular and exosomal circPLEKHM1 expression was notably inhibited by HIF1A knockdown (Figure 1I, and Figure S1N,O, Supporting Information). These results suggested that exosomal circPLEKHM1 in NSCLC was induced by HIF1A under hypoxic conditions.

Notably, in a clinical cohort of 110 human tumors from NSCLC patients, significantly higher levels of circPLEKHM1 were observed in tumors from patients with bone metastasis than those without metastasis, despite the fact that the expression of circPLEKHM1 in tumors was comparable to that in their adjacent tissue (Figure 1J,K). Importantly, considering the age, sex, and stage factors, multivariable logistical regression analysis revealed that high tumor circPLEKHM1 levels were a significant risk factor for lung cancer metastasis, which is consistent with the results of univariable logistical regression analysis (Table S1, Supporting Information). Furthermore, the circPLEKHM1 expression was significantly associated with HIF1A expression in NSCLC patients (Figure 1L). These findings suggest that circPLEKHM1 was upregulated by HIF1A upon hypoxia in human tumor samples, and circPLEKHM1 might contribute to NSCLC metastasis under hypoxia.

### 2.3 Hypoxic Exosomes Promote Lung Cancer Migration Requiring Macrophages

To explore the role of hypoxia‐induced exosomes in NSCLC metastasis, we first investigated whether these exosomes can directly affect the migration of NSCLC cells. Normoxic or hypoxic exosomes were co‐cultured with the A549 or PC9 NSCLC cells, and transwell assays were performed to detect NSCLC cell migration (Figure S2A, Supporting Information). Unexpectedly, only a mild increase in tumor cell migration was observed in hypoxia‐induced exosomes compared to that in PBS control, and the effect of promoting tumor cell migration was comparable between hypoxic and normoxic exosomes (Figure S2A, Supporting Information). These results indicated that hypoxia‐induced exosomes may play major roles other than directly affecting cancer cells during migration.

**Figure 2:**
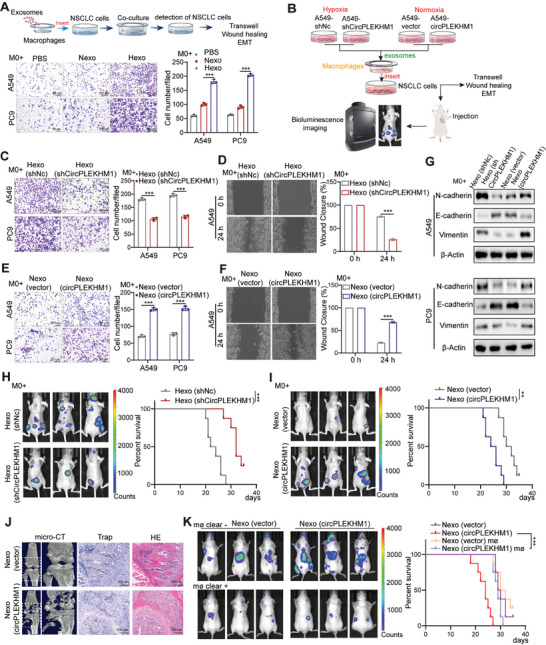
Hypoxia‐induced exosomal circPlEKHM1 promotes NSCLC metastasis through macrophages. A) Schematics of the co‐culture system (upper). Transwell migration assay of A549 and PC9 cells co‐cultured with M0 macrophages treated with indicated exosomes. Scale bar, 50 µm (lower). B) Schematics of the workflow: A549 cells were stably transfected with shCircPLEKHM1 or shNc under hypoxia, circPLEKHM1 or vector under normoxia. Exosomes were collected following the flowchart and co‐cultured with NSCLC cells for subsequent functional experiments. C–F) Transwell migration assay of C) A549 and E) PC9 cells and D,F) wound healing assay of A549 cells co‐cultured with M0 macrophages treated with indicated exosomes. Scale bar, 50 µm. G) A549 and PC9 cells were co‐cultured with M0 macrophages treated with indicated exosomes, and the expression of metastasis‐related proteins was detected by Western blotting. H,I) A549 cells were pre‐co‐cultured with M0 macrophages treated with indicated exosomes and then transplanted into nude mice via intracardiac injection. The growth of bone metastasis was monitored by IVIS Bioimager. Kaplan–Meier analysis was performed for survival, **p < 0.01, ***p < 0.001 by log‐rank test (*n* = 8). J) Legs of mice were imaged by micro‐CT. The TRAP staining was performed in tumor‐bearing bones from mice, with H&E staining of the same field. Scale bar, 100 µm. K) Macrophages were depleted by intraperitoneal injection of clodronate liposomes in mice. Mice were injected with NSCLC cells via intracardiac injection to establish a lung cancer metastasis model and exosomes were administered through the tail vein. The NSCLC metastasis was monitored by IVIS Bioimager. Kaplan–Meier analysis was performed for survival, ***p < 0.001 by log‐rank test (*n* = 8). Data in (A) were presented as mean ± SD, ***p < 0.001 by one‐way ANOVA test. Data in (C–F) are presented as mean ± SD, ***p < 0.001 by Student's* t*‐test.

Tumor stromal cells are indispensable for tumorigenesis and cancer progression. Therefore, we hypothesized that hypoxic exosomes might affect the tumor cell migration by interacting with tumor stromal cells. Using an indirect co‐culture system, normoxic or hypoxic exosomes were first added to the major tumor stromal cells (cancer‐associated fibroblasts, endothelial cells, mesenchymal stem cells, and macrophages) for 48 h. Next, NSCLC cells were separately co‐cultured with the exosome‐educated tumor stromal cells, followed by transwell assays to evaluate cancer cell migration (**Figure** **2**A, and S2B, Supporting Information). Macrophages pretreated with hypoxic exosomes exhibited the most dramatic effect on increasing the number of migratory NSCLC cells (Figure 2A, and Figure S2B, Supporting Information). In addition, macrophages pretreated with hypoxic exosomes promoted NSCLC cell migration and elevated N‐cadherin and vimentin protein levels in NSCLC cells (Figure S2C–E, Supporting Information). These results suggested that hypoxic exosomes could mediate NSCLC cell metastasis via macrophages.

### 2.4 Exosomal circPLEKHM1 Educated Macrophages to Promote Lung Cancer Metastasis In Vitro and In Vivo

Next, we investigated whether exosomal circPLEKHM1 promoted NSCLC metastasis via macrophages. We constructed back‐splicing sequence‐targeting shRNAs to silence circPLEKHM1 and a plasmid carrying circPLEKHM1 to upregulate circPLEKHM1 in exosomes derived from normoxic A549 cells (Figure S2F,G, Supporting Information). Exosomes from these circPLEKHM1‐altered cells were extracted to train macrophages (Figure 2B). Notably, macrophages treated with sh‐circPLEKHM1 exosomes (Hexo shCircPLEKHM1) showed significantly reduced migration and invasion of co‐cultured A549 and PC9 cells compared to those educated with control (Hexo shNc) (Figure 2C,D, and Figure S2H, Supporting Information). In contrast, macrophages exposed to exosomes overexpressing circPLEKHM1 (Nexo circPLEKHM1) showed remarkably increased migration and invasion of co‐cultured A549 and PC9 cells, compared to the control (Nexo vector) (Figure 2E,F, and Figure S2I, Supporting Information). Moreover, the protein levels of N‐cadherin and vimentin in NSCLC cells were significantly suppressed after co‐cultured with Hexo shCircPLEKHM1 educated macrophages, but were enhanced after co‐cultured with Nexo circPLEKHM1 educated macrophages (Figure 2G). These results suggested that exosomal circPLEKHM1 could train macrophages to promote NSCLC cell metastasis.

To further explore the role of hypoxia‐induced exosomal circPLEKHM1 in educating macrophages to regulate NSCLC metastasis in vivo, luciferase labeled A549 cells were co‐cultured with circPLEKHM1‐altered exosomes pretreated macrophages, followed by intracardiac injection to establish a mouse tumor metastasis model (Figure 2B). Interestingly, bioluminescence imaging revealed that the number of metastatic lesions was significantly reduced in mice intracardially injected with NSCLC A549 cells co‐cultured with hypoxic shCircPLEKHM1 exosome pre‐trained macrophages (Hexo shCircPLEKHM1 group) than in the control mice group (Hexo shNC group) (Figure 2H, and Figure S2J, Supporting Information). Moreover, the survival of mice in the Hexo shCircPLEKHM1 group was significantly longer than that of the Hexo shNC group (Figure 2H). In contrast, a significantly increased number and size of metastatic lesions were observed in mice intracardially injected with A549 cells co‐cultured with macrophages trained by normoxic circPLEKHM1 overexpressed exosome (Nexo circPLEKHM1 group) compared with the control group (Figure 2I, and Figure S2K, Supporting Information). Consistently, the survival of the Nexo circPLEKHM1 group mice was significantly shorter than that of the control group (Figure 2I). Furthermore, micro‐CT scans revealed that the mice in the Nexo circPLEKHM1 group experienced robustly increased bone destruction, as shown by a notable decrease in bone density and osteolytic destruction in bone metastatic lesions (Figure 2J). These findings demonstrated that the overexpression of exosomal circPLEKHM1, which coordinates with macrophages, causes more severe bone metastasis in NSCLC. Collectively, exosomal circPLEKHM1‐educated macrophages significantly promoted NSCLC metastasis in vivo.

To further investigate whether exosomal circPLEKHM1‐mediated lung cancer metastasis occurs through macrophages, we treated mice with clodronate liposomes to deplete macrophages in vivo. Strikingly, macrophage depletion dramatically abolished the effect of exosomal circPLEKHM1 on promoting lung cancer metastasis in vivo. The robust increase in lung cancer metastasis by exosomes overexpressing circPLEKHM1 in pretreated macrophages was significantly reduced, and mice survival was improved after clodronate liposomes treatment (Figure 2K, and Figure S2L, Supporting Information). These results demonstrated that macrophages are required for NSCLC metastasis mediated by exosomal circPLEKHM1. Taken together, hypoxia‐induced exosomal circPLEKHM1 promoted the metastasis of NSCLC through macrophages.

### 2.5 Hypoxia‐Induced Exosomal circPLEKHM1 Induces M2 Macrophage Polarization

**Figure 3:**
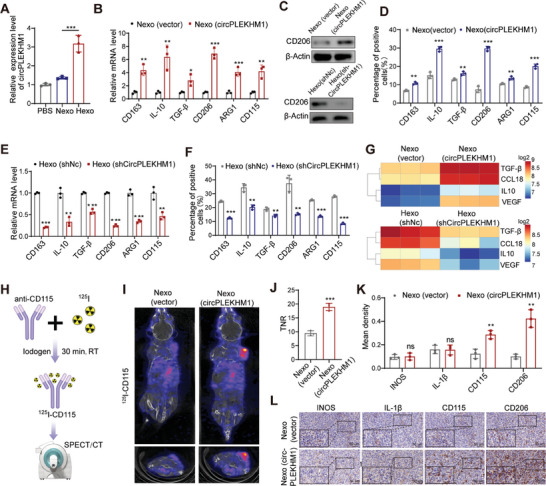
Exosomal circPLEKHM1 induces macrophage M2‐type polarization in vitro and in vivo. A) Expression of circPLEKHM1 in macrophages treated with indicated exosomes. B) RT‐qPCR assay for *CD163*, *IL‐10*, *TGF‐β*, *CD206*, *ARG1*, and *CD115* mRNA levels in macrophages treated with Nexo (circPLEKHM1). Western blot analysis to detect protein expression of CD206 in macrophages treated with indicated exosomes. Flow cytometry staining CD163, IL‐10, TGF‐β, CD206, ARG1, and CD115 to analyze the effect of Nexo (circPLEKHM1) on macrophages. E) RT‐qPCR assay and F) flow cytometry staining for CD163, IL‐10, TGF‐β, CD206, ARG1, and CD115 to analyze the effect of Hexo (shCircPLEKHM1) on macrophages. G) Multiplex immunoassay to detect secreted cytokines by macrophages treated with indicated exosomes. H) Schematic diagram of the synthesis of radionuclide ^125^I‐CD115 probe for micro‐SPECT/CT to detect CD115 in vivo. I) Representative image of micro‐SPECT/CT in mice bearing tumors treated with indicated exosomes via intratumoral injection, followed by a single intravenous injection of ^125^I‐CD115 before micro‐SPECT/CT scan. J) Target‐to‐normal tissue ratio (TNR) of micro‐SPECT/CT scan in the indicated mouse groups. K,L) IHC assays for INOS, IL‐1β, CD115, and CD206 in tumor tissues. Scale bar, 50 µm. Data in (A) were presented as mean ± SD, ***p < 0.001 by one‐way ANOVA test. Data in (B,D–F) were presented as mean ± SD, *p < 0.05, **p < 0.01, ***p < 0.001 by two‐tailed Student's* t*‐test. Data in (J,K) were presented as mean ± SD, *p < 0.05, **p < 0.01, ***p < 0.001 by non‐parametric Mann–Whitney test.

As we previously reported, hypoxic exosomes promote M2‐type macrophage polarization to promote tumor malignancy.^[^ ^26^ ^]^ Therefore, we investigated whether exosomal circPLEKHM1 affected macrophage polarization during hypoxia. By labeling exosomes with PKH26, both hypoxia‐ and normoxia‐induced exosomes were internalized by M0 macrophages (Figure S3A, Supporting Information). Notably, the level of circPLEKHM1 was significantly higher in M0 macrophages incubated with hypoxia‐induced exosomes than in those incubated with normoxia‐induced exosomes (**Figure** **3**A), indicating that hypoxia‐induced exosomal circPLEKHM1 can be transferred to M0 macrophages. Besides, hypoxia‐induced exosomes derived from NSCLC cells boosted the expression of the M2 markers (CD163, IL‐10, TGF‐β, CD206, ARG1, and CD115) in macrophages, but not M1 markers (IL‐1β and INOS) (Figure S3B–D, Supporting Information), indicating a polarization toward M2‐type macrophage. Furthermore, exosomes secreted by circPLEKHM1 overexpressed NSCLC cells under normoxia (Nexo‐circPLEKHM1) caused a significant increase in the mRNA expression of multiple M2 markers (CD163, IL‐10, TGF‐β, CD206, ARG1, or CD115), protein levels of CD206, and proportion of M2 markers positive cells in macrophages, but not mRNA or protein expression of M1 markers (IL‐1β, INOS, CD86) (Figure 3B–D, and Figure S3E–G, Supporting Information). In contrast, exosomes secreted by circPLEKHM1‐knockdown NSCLC cells under hypoxia (Hexo‐shCircPLEKHM1) dramatically decreased M2 markers expression in macrophages and the percentage of M2‐type macrophages, but had little effect on M1‐type markers (Figure 3C,E,F, and Figure S3H–J, Supporting Information). Lastly, multiplex immunoassay showed that circPLEKHM1 overexpressed exosomes notably enhanced the secretion of TGF‐β, CCL18, IL10, and VEGF, which are primarily associated with M2 macrophages (Figure 3G). Conversely, treatment with Hexo shCircPLEKHM1 exosomes resulted in a significant reduction in the secretion of M2‐type cytokines by macrophages (Figure 3G). These results suggested that exosomal circPLEKHM1 can induce macrophage polarization toward to M2‐type direction.

### 2.6 ^125^I‐CD115 Micro‐SPECT/CT Molecular Imaging Reveals Exosomal circPLEKHM1 can Induce M2 Polarization of Tumor‐Associated Macrophage In Vivo

To further explore the impact of exosomal circPLEKHM1 on macrophage polarization in vivo, we generated a new radioactive molecular probe for single‐photon emission computed tomography/computed tomography (SPECT/CT) imaging to detect whole‐body M2 type of macrophage in vivo, by labeling a monoclonal antibody targeting the M2 marker CD115 with radionuclide ^125^I (Figure 3H). The initial labeling yield of ^125^I‐CD115 was 99.99% (Figure S3K, Supporting Information). During the 72‐h incubation of ^125^I‐CD115 with PBS or FBS, the labeling yield was maintained at more than 85% (Figure S3L, Supporting Information), indicating that ^125^I‐CD115 was a stable molecular probe for SPECT/CT imaging. The biodistribution map of ^125^I‐CD115 in tumor‐bearing mice was obtained by measuring the radioactivity levels in various organ tissues of the sacrificed mice at different time points (Figure S3M, Supporting Information). Notably, the accumulation of ^125^I‐CD115 in tumor tissues was significantly higher than that in other organs (Figure S3M, Supporting Information). In a nutshell, we have developed a reliable molecular tool for detecting tumor‐associated M2 macrophages in vivo.

Importantly, our new ^125^I‐CD115 SPECT/CT imaging of lung cancer‐bearing mice revealed that intra‐tumoral injection of exosomal circPLEKHM1 significantly increased the tumor uptake of ^125^I‐CD115 in vivo (Figure 3I,J). These results further demonstrated that exosomal circPLEKHM1 can aggregate M2 macrophages within the tumor in vivo, as evidenced by the high expression of CD115. In addition, IHC showed that exosomal circPLEKHM1 significantly increased the expression of M2 markers CD206 and CD115 while showing little effect on M1 markers IL‐1β and INOS (Figure 3K,L). Taken together, exosomal circPLEKHM1 can induce intratumoral M2 polarization of macrophages in vivo.

### 2.7 circPLEKHM1 Promotes M2 Polarization of Macrophages by Upregulating OSMR Expression

**Figure 4:**
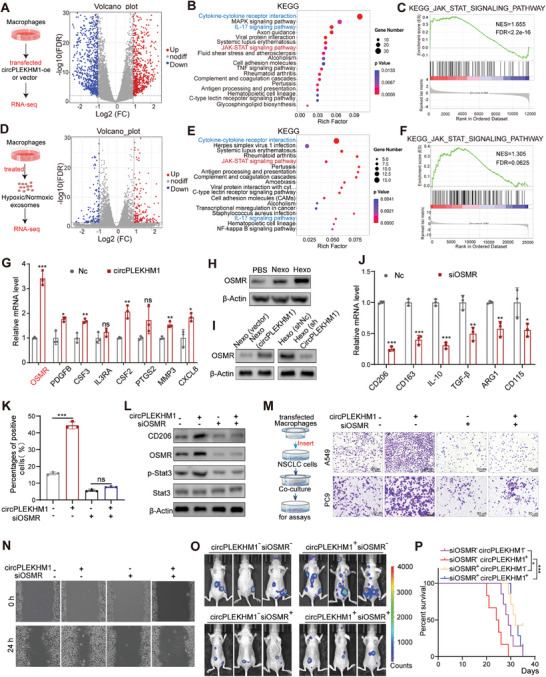
circPLEKHM1 promotes macrophage M2 polarization by OSMR/JAK/STAT axis. A) Volcano plot of mRNA transcript abundance of DEGs in macrophages overexpressed circPLEKHM1 as compared to control (circPLEKHM1‐oe vs control) analyzed by RNA‐seq. *P* < 0.05, log2 (fold change) > 1. B) KEGG pathway enrichment analysis of DEGs (circPLEKHM1‐oe vs control). C) Gene Set Enrichment Analysis (GSEA) for JAK/STAT signaling pathway of DEGs (circPLEKHM1‐oe vs Control). D) Volcano plot of mRNA transcript abundance of DEGs in macrophages treated with hypoxic exosomes as compared to normoxic exosomes (hypoxic vs normoxic exosomes) analyzed by RNA‐seq. *p* <0.05, log2 (fold change) >1. E) KEGG pathway enrichment analysis of DEGs (hypoxic vs normoxic exosomes). F) Gene Set Enrichment Analysis (GSEA) for JAK/STAT signaling pathway of DEGs (hypoxic exosomes versus normoxic exosomes). G) Validation of enriched JAK/STAT and IL‐17 signaling‐related genes in macrophages overexpressing circPLEKHM1 using RT‐qPCR. H‐I) Immunoblot analysis for OSMR in macrophages treated with indicated exosomes. J) RT‐qPCR assay for *CD163*, *IL‐10*, *TGF‐β*, *CD206*, *ARG1*, and *CD115* mRNA levels in macrophages treated with IL‐4 following transfected with siOSMR or control. K) Flow cytometry to detect the expression of CD206 in the treated macrophages. L) Western blot analysis for OSMR, CD206, Stat3, and phosphorylation Stat3 in cells transfected with circPLEKHM1 or siOSMR. M) Transwell migration assay of A549 and PC9 cells co‐cultured with IL4‐treated macrophages transfected with circPLEKHM1 or siOSMR. Scale bar, 50 µm. N) Wound healing assay of A549 cells co‐cultured with IL4‐treated macrophages transfected with circPLEKHM1 or siOSMR. O) A549 cells were co‐cultured with IL4‐treated macrophages transfected with circPLEKHM1 or siOSMR and then transplanted into nude mice via intracardiac injection. The tumor metastasis was monitored by IVIS Bioimager. P) Kaplan–Meier analysis was performed for survival, *p < 0.05, ***p < 0.001 by log‐rank test (*n* = 8). Data in (G,J) were presented as mean ± SD, *p < 0.05, **p < 0.01, ***p < 0.001 by two‐tailed Student's *t*‐test. Data in (K) were presented as mean ± SD, ***p < 0.001 by one‐way ANOVA test.

To elucidate the potential mechanisms underlying the biological effects of circPLEKHM1 on macrophages, RNA‐seq was performed on M0 macrophages overexpressing circPLEKHM1 or vector control (circPLEKHM1‐oe vs control). There were 515 upregulated and 721 downregulated genes differentially expressed in circPLEKHM1 overexpressed macrophages (circPLEKHM1‐oe vs control) (**Figure** **4**A). The GO analysis on the transcriptome data revealed that circPLEKHM1 impacted multiple macrophage‐involved functions including immune response and cytokine activity (Figure S4A, Supporting Information). Moreover, the KEGG pathway enrichment analysis showed that overexpression of circPLEKHM1 in macrophages caused significant alterations in several pathways related to M2 macrophage polarization, such as cytokine–cytokine receptor interaction, axon guidance, the IL‐17 signaling pathway, and the JAK‐STAT signaling pathway (Figure 4B). GSEA analysis further revealed that multiple M2‐macrophage‐involved pathways were enriched in circPLEKHM1 overexpressed macrophages (Figure 4C, and Figure S4B, Supporting Information). These results further supported the notion that circPLEKHM1 is closely related to macrophage M2 polarization. Intriguingly, in another RNA‐Seq analysis investigating the transcriptome alterations of macrophages by hypoxia‐induced exosomes as compared to normoxia exosomes (hypoxic exosomes vs normoxic exosomes), we also observed significant alterations in multiple pathways associated with M2 macrophage polarization, such as cytokine‐cytokine receptor interaction, the IL‐17 signaling pathway, and the JAK‐STAT signaling pathway (Figure 4D–F). The concurrent enrichment of the JAK‐STAT pathway and other M2‐macrophage‐related pathways identified in macrophages by circPLEKHM1 overexpression or hypoxia‐induced exosomes. Additionally, GSEA similarity network analysis indicated that multiple interleukin‐related pathways and the KEGG cytokine–cytokine receptor interaction pathway associated with the JAK‐STAT pathway were significantly enriched (Figure S4C, Supporting Information), further supporting that circPLEKHM1 enriched in hypoxic exosomes might regulate the macrophage polarization by JAK‐STAT pathway.

Next, we treated circPLEKHM1 overexpressing macrophages with the JAK‐STAT3 pathway inhibitor SC99 to investigate whether circPLEKHM1 regulates macrophage polarization through the JAK‐STAT3 pathway. While overexpression of circPLEKHM1 markedly enhanced the p‐STAT3 level in macrophages, SC99 treatment blunted the circPLEKHM1‐upregulated p‐STAT3 level in macrophages (Figure S4D, Supporting Information). Importantly, SC99 significantly suppressed circPLEKHM1‐induced M2 polarization in macrophages (Figure S4E, Supporting Information), supporting that circPLEKHM1 regulates macrophage polarization via the JAK‐STAT pathway.

Notably, OSMR, an important regulator of the JAK‐STAT3 signaling pathway, was the gene most significantly upregulated by circPLEKHM1‐overexpression in macrophages (Figure 4G). Therefore, we hypothesized that circPLEKHM1 is involved in the regulation of the JAK‐STAT3 pathway by upregulating OSMR expression. Indeed, Western blotting showed a higher expression of OSMR in macrophages treated with hypoxic exosomes than in those treated with normoxic exosomes (Figure 4H). Moreover, OSMR expression was enhanced when macrophages were co‐cultured with normoxic exosomes overexpressing circPLEKHM1 (Figure 4I). Consistent with this, OSMR expression was suppressed when macrophages were co‐cultured with exosomes treated with shcircPLEKHM1 under hypoxic conditions (Figure 4I). Moreover, the GEPIA database^[^ ^27^ ^]^ showed that the expression level of OSMR was significantly higher in M2 macrophages than in M1 or M0 macrophages (Figure S4F, Supporting Information). Overall, exosomal circPLEKHM1 could robustly upregulate OSMR expression in macrophages.

Next, we investigated whether exosomal circPLEKHM1 regulates macrophage M2 polarization through OSMR. Indeed, the knockdown of OSMR in M2 macrophages (IL‐4‐treated M0 macrophages) significantly inhibited the expression of the M2 markers in macrophages (Figure 4J, and Figure S4G, Supporting Information). Moreover, when we knocked down OSMR based on circPLEKHM1 overexpression, circPLEKHM1 lost its ability to promote macrophage M2 polarization upon OSMR depletion, as measured by the rescued CD206 positive macrophages and CD206 expression (Figure 4K,L, and Figure S4H, Supporting Information). Finally, OSMR knockdown reversed the circPLEKHM1‐upregulated expression of phospho‐Stat3 (p‐Stat3) (Figure 4L). These findings indicated that circPLEKHM1 requires OSMR to regulate the JAK‐STAT3 pathway and promote macrophage M2 polarization.

### 2.8 circPLEKHM1 Requires OSMR to Facilitate M2 Macrophage‐Mediated Lung Cancer Metastasis

Next, we investigated whether OSMR is critical for circPLEKHM1 in mediating M2 macrophage‐mediated lung cancer metastasis. We knocked down OSMR based on circPLEKHM1 overexpression in M2 macrophages induced from M0 macrophages with IL‐4, co‐cultured these M2 macrophages with A549 and PC9 NSCLC cells, and then conducted transwell assays to detect NSCLC cell migration (Figure 4M). Interestingly, while circPLEKHM1 overexpression in macrophages significantly increased the migration and invasion of both A549 and PC9 cells, OSMR knockdown dramatically blunted the effects of circPLEKHM1 overexpression in promoting NSCLC cells migration (Figure 4M,N, and Figure S4I,J, Supporting Information). These results suggested OSMR was essential for circPLEKHM1 to promote M2 macrophage‐associated lung cancer metastasis.

To further investigate the role of OSMR in circPLEKHM1‐mediated lung cancer metastasis in vivo, a mouse tumor metastasis model was established by intracardially injecting the A549 cells co‐cultured with circPLEKHM1 and/or OSMR‐altered macrophages. Impressively, knockdown of OSMR significantly inhibited circPLEKHM1‐enhanced lung cancer metastasis in vivo and rescued metastatic lesions, as measured by bioluminescence imaging and mice overall survival (Figure 4O,P, and Figure S4K, Supporting Information). Collectively, circPLEKHM1 induced M2 macrophage to promote lung cancer metastasis via OSMR.

### 2.9 circPLEKHM1 Interacting with PABPC1 Stabilizes OSMR mRNA and Promotes its Expression

**Figure 5:**
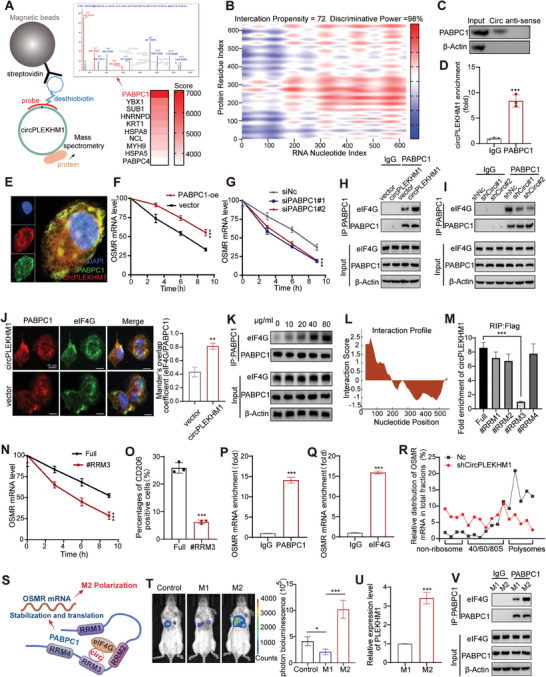
circPLEKHM1/PABPC1/eIF4G RNA‐protein ternary complex stabilizes OSMR mRNA and promotes its expression to enhance M2 macrophage polarization. A) Schematic illustration of identification potential circPLEKHM1‐associated proteins using RNA antisense purification assays and LC‐MS/MS analysis. B) Prediction of the binding degree of circPLEKHM1 and PABPC1 using the RAPID database. C) Western blot analysis of PABPC1 derived from RNA pull‐down using the circPLEKHM1 probe. D) RT‐qPCR analysis of circPLEKHM1 derived from RIP assay with anti‐PABPC1 antibody. E) Localization of circPLKEHM1 and PABPC1 by FISH and IF. Scale bar, 50 µm. F,G) After being treated with actinomycin D, total RNA was extracted from cells transfected with F) PABPC1 or G) siPABPC1 at different times for RT‐qPCR. H,I) Co‐immunoprecipitation assay to pull down PABPC1 to detect the interaction between eIF4G and PABPC1 in cells transfected with cirPLEKHM1 or shirPLEKHM1. J) Immunofluorescence showed co‐localization of eIF4G and PABPC1 in cells was enhanced by transfection of cirPLEKHM1. K) Interaction between eIF4G and PABPC1 in cells treated with the indicated dose of hypoxic exosomes. L) Prediction of the binding region of circPLEKHM1 and PABPC1 using the RAPID database. M) RT‐qPCR analysis of RNAs derived from RIP assay with anti‐FLAG antibody. N) After being treated with actinomycin D, total RNA was extracted from cells transfected with PABPC1‐WT or PABPC1‐RRM3# at different times for RT‐qPCR. O) Flow cytometry to detect the CD206 positive cell percentages in the indicated macrophages. P) RT‐qPCR analysis of OSMR derived from RIP assay with anti‐PABPC1 antibody. Q) RT‐qPCR analysis of OSMR derived from RIP assay with anti‐eIF4G antibody. R) Polysome profiling coupled with RT‐qPCR analysis of cells with circPLEKHM1 knockdown of OSMR mRNA distribution in different ribosome fractions. S) Illustration of circPLEKHM1 enhancing the interaction between eIF4G and PABPC1, and subsequently promoting OSMR mRNA stabilization and translation. T) M1 and M2 macrophages were tail vein injected with LA795 lung cancer cells in mice. The lung cancer metastasis was monitored by IVIS Bioimager. U) RT‐qPCR analysis of circPLEKHM1 in M1 and M2 macrophages derived from tumor tissues. V) Co‐immunoprecipitation assay to pull down PABPC1 to detect its interaction with eIF4G in M1 and M2 macrophages derived from tumor tissues. Data in (D,J,O—Q,U) were presented as mean ± SD, **p < 0.01, ***p < 0.001 by two‐tailed Student's *t*‐test. Data in (F,G,N) were presented as mean ± SD, ***p < 0.001 by two‐way ANOVA test. Data in (M,T) were presented as mean ± SD, *p < 0.05, ***p < 0.001 by one‐way ANOVA test.

CircRNAs can act as protein sponges by interacting with RBPs, which are key players in regulating gene expression.^[^ ^28^ ^]^ This interaction enables circRNAs to mediate physiological and pathological functions.^[^ ^21^ ^]^ To further investigate how circPLEKHM1 modulates OSMR expression, mass spectrometry was performed using the products obtained from the RNA pull‐down to identify potential circPLEKHM1‐associated proteins (**Figure** **5**A). Among the proteins enriched by the circPLEKHM1‐sense probe, PABPC1 was a top abundant candidate and was predicted to have the strongest binding among the top three abundant proteins (Figure 5A,B). In addition, RNA pull‐down, followed by western blotting showed that PABPC1 was precipitated by the biotin‐labeled circPLEKHM1 probe, which was further confirmed via RNA immunoprecipitation (RIP) (Figure 5C,D). In addition, immunofluorescence analysis indicated that circPLEKHM1 colocalized with PABPC1 (Figure 5E). These results suggested that PABPC1 protein is a strong interactor of circPLEKHM1.

PABPC1 binds to the poly (A) tail of mRNA and promotes mRNA stability and translation.^[^ ^29^, ^30^ ^]^ Thus, we asked whether PABPC1 is involved in modulating OSMR expression. Indeed, when treated with actinomycin D, the degradation of OSMR mRNA significantly decelerated in PABPC1‐overexpressed macrophages (Figure 5F). In contrast, the degradation of OSMR mRNA was accelerated when PABPC1 was silenced by siRNA (Figure 5G). Consistently, the protein level of OSMR was significantly increased when PABPC1 was overexpressed and decreased when PABPC1 was silenced (Figure S5A, Supporting Information). Taken together, these results suggest that circPLEKHM1 might regulate OSMR expression by interacting with PABPC1.

### 2.10 circPLEKHM1/PABPC1/eIF4G RNA‐Protein Ternary Complex Enhances OSMR Translation and M2 Macrophage Polarization

Next, we investigated how circPLEKHM1 interacts with PABPC1 to promote OSMR expression. Unexpectedly, neither gain nor loss of circPLEKHM1 expression dramatically affected the expression of PABPC1 (Figure S5B, Supporting Information), suggesting that circPLEKHM1 was unlikely to regulate PABPC1 expression. PABPC1 plays a key role in increasing mRNA stability and promoting translation initiation by interacting with eIF4G, an essential component of the cap‐binding complex.^[^ ^31^, ^32^, ^33^ ^]^ Interestingly, eIF4G was one of the RBPs assigned to circPLEKHM1 identified by mass spectrometry and further confirmed by RIP (Figure S5C, Supporting Information), suggesting that circPLEKHM1 might affect the PABPC1‐eIF4G interaction to regulate OSMR expression. Notably, the PABPC1‐eIF4G interaction was markedly increased when circPLEKHM1 was overexpressed (Figure 5H) and was decreased by the knockdown of circPLEKHM1 in macrophages (Figure 5I). Moreover, circPLEKHM1 significantly promoted the fluorescence co‐localization of PABPC1 and eIF4G (Figure 5J). In addition, adding hypoxic exosomes that enriched circPLEKHM1 promoted the PABPC1‐eIF4G interaction in a concentration‐dependent manner (Figure 5K, and Figure S5D, Supporting Information). All these results suggested the enhanced PABPC1‐eIF4G interaction by circPLEKHM1.

The eIF4G has been reported to directly bind to PABPC1 in the RRM2 domain; however, its binding affinity is weak and requires the assistance of two adjacent domains of PABPC1, RRM1, and RRM3.^[^ ^32^ ^]^ We used the catRAPID website to predict the potential binding regions of PABPC1‐circPLEKHM1 and found that the binding regions of PABPC1 may be A101‐152, A201‐252, and A236‐287 (Figure 5L), which are located at RRM2 and RRM3. To identify the location of PABPC1 binding to circPLEKHM1, we constructed five flag‐labeled PABPC1‐mutant protein plasmids and tested their binding ability with circPLEKHM1 (Figure S5E,F, Supporting Information). Notably, the RIP‐qPCR results showed that the interaction between PABPC1‐RRM3 and circPLEKHM1 was significantly decreased compared to the interaction between PABPC1‐full and circPABPC1 (Figure 5M). These results suggested that the RRM3 of PABPC1 was critical for its interaction with circPLEKHM1. Moreover, compared to wild‐type PABPC1, RRM3‐mutant PABPC1 significantly decreased the stability and expression of OSMR in circPLEKHM1 overexpressed macrophages (Figure 5N, and Figure S5G, Supporting Information). Furthermore, compared with wild‐type PABPC1, RRM3‐mutant PABPC1 dramatically reduced the M2 polarization of macrophages induced by circPLEKHM1 (Figure 5O, and Figure S5H, Supporting Information). These results suggested that circPLEKHM1 interacted with PABPC1‐RRM3 to regulate the OSMR gene and enhance M2 macrophage polarization.

We further attempted to investigate how the CircPLEKHM1/PABPC1/eIF4G RNA‐protein ternary complex recognizes OSMR mRNA and enhances its expression. RNA immunoprecipitation (RIP) experiments showed that circPLEKHM1 did not bind to OSMR mRNA (Figure S5I, Supporting Information). Instead, both PABPC1 and eIF4G could directly bind to OSMR mRNA (Figure 5P,Q). PABPC1 exhibits high‐affinity binding to adenylate‐rich sequences in mRNA and the interaction of PABPC1 with the poly(A) tail activates translation initiation.^[^ ^34^, ^35^ ^]^ Our findings suggested that PABPC1 could interact with OSMR mRNA, primarily binding to the 3′ OSMR region, supporting the mechanism of PABPC1 binding to poly(A) (Figure 5P, and Figure S5J, Supporting Information). Conversely, eIF4G bound to OSMR mRNA, predominantly associating with the 5′ OSMR region (Figure 5Q, and Figure S5K, Supporting Information). EIF4G is a component of the eIF4F protein complex that is involved in recognizing mRNA cap structures, unwinding 5′‐terminal secondary structures in an ATP‐dependent manner, and facilitating the recruitment of mRNA to the ribosome for translation initiation.^[^ ^35^ ^]^ These findings suggested that circPLEKHM1 functioned as an important scaffold for the PABPC1‐eIF4G interaction to regulate OSMA mRNA translation. In addition, we performed a polysome profiling analysis and indicated that circPLEKHM1 knockdown leads to a reduction in translational activation. This reduction is primarily attributed to a substantial decrease in the polysome content within circPLEKHM1 knockdown cells (Figure S5L, Supporting Information). Notably, circPLEKHM1 knockdown caused a significant shift of OSMR mRNA to the non‐polysome fractions, along with a reduction in OSMR mRNA levels in the translation fractions, suggesting diminished OSMR translational activity after circPLEKHM1 knockdown (Figure 5R). Taken together, our findings support a molecular mechanism by which circPLEKHM1 functions as an important scaffold for the PABPC1‐eIF4G interaction, further promoting OSMA mRNA translation (Figure 5S).

Lastly, to confirm the vital role of this circPLEKHM1/PABPC1/eIF4G complex in regulating macrophage polarization in vivo, we detected this complex in macrophages isolated from lung cancer metastases in a mice model established through the tail vein injection of LA795 lung cancer cells. We observed a notable increase in lung cancer metastasis when LA795 cells were administrated with M2 macrophages rather than administrated with the M1 macrophages group or control group (Figure 5T). Then, we isolated M1 and M2 macrophages from lung cancer metastases in this control mice group. Notably, RT‐qPCR analysis showed significantly higher expression levels of circPLEKHM1 in tumor‐infiltrated M2 macrophages than in M1 macrophages (Figure 5U). Immunoprecipitation assays further revealed a much stronger interaction between PABPC1 and eIF4G in tumor‐infiltrated M2 macrophages as compared to M1 macrophages (Figure 5V). Taken together, our findings suggested that circPLEKHM1/PABPC1/eIF4G RNA‐protein ternary complex promoted the OSMR translation, enhancing M2 macrophage polarization to promote lung cancer metastasis (Figure 5S).

### 2.11 Targeting Exosomal circPLEKHM1 Shows Significant Therapeutic Benefits in Treating NSCLC Metastasis In Vivo

**Figure 6:**
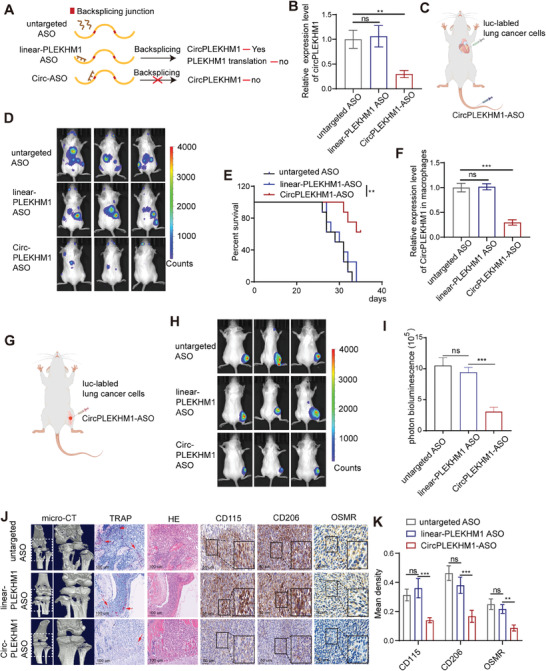
circPLEKHM1‐targeted therapy effectively reduced NSCLC metastases in vivo. A) Design of nucleic acid drugs targeting circPLEKHM1 or non‐circle region of linear RNA of PLEKHM1. B) Validation of the knockdown effect of circPLEKHM1‐ASO in A549 cells. C) Schematic illustration of lung cancer metastasis model that lung cancer cells were transplanted into mice via intracardiac injection and then intravenous injection of circPLEKHM1‐ASO. D) Metastasis of mice was monitored by IVIS Bioimager. E) Kaplan–Meier analysis was performed for survival, **p < 0.01 by log‐rank test (*n* = 8). F) circPLEKHM1‐ASO decreased circPLEKHM1 levels in metastases infiltrated macrophages. G) Schematic illustration of NSCLC bone metastasis model that lung cancer cells were transplanted into mice via tibial plateau injection and then intravenous injection of circPLEKHM1‐ASO. H,I) Bone metastasis of mice was monitored by IVIS Bioimager. J,K) Legs of mice were imaged by micro‐CT. The TRAP staining was performed in tumor‐bearing bones from mice and H&E staining of the same field was shown together with the TRAP staining. Scale bar, 100 µm. IHC assays for CD115, CD206, and OSMR of tumor tissues. J) Scale bar, 50 µm. K) Quantification of IHC assays for CD115, CD206, and OSMR of tumor tissues. Data in (B,F,I,K) were presented as mean ± SD, **p < 0.01, ***p < 0.001 by one‐way ANOVA test.

Our observation that exosomal circPLEKHM1‐induced polarization of macrophages promotes NSCLC metastasis, led us to investigate the therapeutic potential of targeting circPLEKHM1 for the treatment of NSCLC metastasis. Using antisense oligonucleotide targeting the circPLEKHM1‐specific back‐splicing sequence (circPLKHM1‐ASO), we successfully reduced the levels of circPLEKHM1 with no significant impact on the levels of either linear PLEKHM1 or PLEKHM1 mRNA in A549 cells (**Figure** **6**A,B, and S5M, Supporting Information). To investigate the effect of circPLEKHM1‐ASO in vivo, we administered circPLEKHM1‐ASO to a mouse model of lung cancer with tumor metastasis via intracardiac injection (Figure 6C). Strikingly, circPLEKHM1‐ASO therapy dramatically reduced lung cancer metastasis and increased the overall survival rate compared with the control (Figure 6D,E), along with significantly decreased circPLEKHM1 levels in metastases infiltrated macrophages (Figure 6F). In another preclinical mouse model of bone metastasis caused by lung cancer (Figure 6G), we obtained consistent results that circPLEKHM1‐ASO therapy dramatically decreased bone metastasis of NSCLC (Figure 6H,I). Moreover, an in vivo micro‐CT scan showed that circPLEKHM1‐ASO therapy significantly reduced bone destruction and increased bone density during bone metastasis of NSCLC‐bearing mice (Figure 6J). Consistently, a significant decrease in M2 macrophages markers (CD206 and CD115) expression and OSMR expression were found in the tumors of mice treated with circPLEKHM1‐ASO therapy (Figure 6J,K), supporting our notion that circPLEKHM1 promotes metastasis of NSCLC by regulating OSMR mediated macrophages polarization. Collectively, circPLEKHM1‐ASO significantly inhibited NSCLC metastasis and decreased tumor M2‐type macrophages in vivo.

Notably, unlike the effect of circPLEKHM1‐silencer on reducing NSCLC metastasis, knockdown of non‐circPLEKHM1 sequence region of the host gene PLEKHM1 (linear‐PLEKHM1 ASO) showed no significant effect in reducing metastasis or survival of NSCLC both in the metastasis model of lung cancer via intracardiac injection and model of bone metastasis (Figure 6D–I). Moreover, linear‐PLEKHM1 ASO did not reduce the M2 markers of macrophages and OSMR expression in metastatic NSCLC tumors (Figure 6J,K). These results indicated that circPLEKHM1, rather than the noncircular region of its host linear PLEKHM1, is a critical therapeutic target for inhibiting NSCLC metastasis via OSMR‐mediated M2 macrophage.

### 2.12 Clinical Relevance of the circPLEKHM1/OSMR/M2 Macrophage Polarization Axis in NSCLC Patients

**Figure 7:**
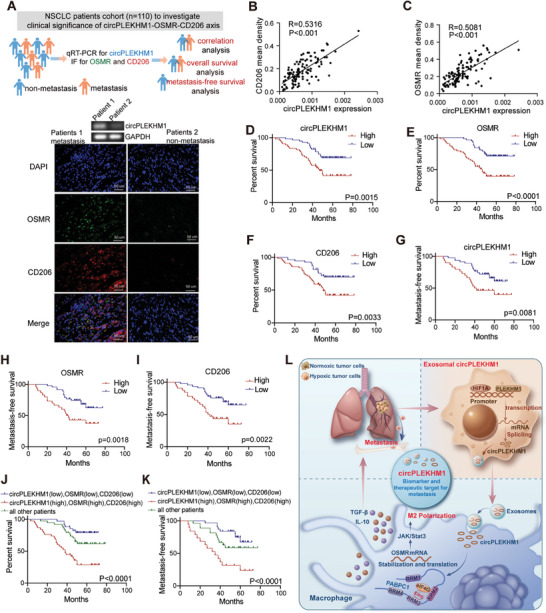
circPLEKHM1‐OSMR‐CD206 axis functions as significant biomarkers for metastasis and prognosis in NSCLC patients. A) Schematic diagram of NSCLC patient samples (upper). circPLEKHM1, CD206, and OSMR expression levels in representative patients with NSCLC are shown (lower). Scale bar, 50 mm. B,C) Scatter plots depicting the associations between circPLEKHM1 and CD206 or OSMR in tumor tissues from NSCLC patients. D–F) Kaplan–Meier analysis to determine the prognostic values of circPLEKHM1 or CD206 or OSMR for overall survival in NSCLC patients, *p*‐value by log‐rank test (*n* = 110). G–I) Kaplan–Meier analysis to determine the prognostic values of circPLEKHM1 or CD206 or OSMR for metastasis‐free survival in NSCLC patients, *p*‐value by log‐rank test (*n* = 77). J,K) Kaplan–Meier analysis to determine the prognostic values of circPLEKHM1‐CD206‐OSMR axis for patient overall survival (*n* = 110) and metastasis‐free survival (*n* = 77), *p*‐value by log‐rank test. L) Graphical illustration of how NSCLC‐secreted exosomal circPLEKHM1 that induced under hypoxia mediates the tumor cell‐macrophage communication to promote metastasis. circPLEKHM1 functions as a biomarker and therapeutic target for NSCLC metastasis.

To investigate the clinical significance of the circPLEKHM1/OSMR/M2 macrophage axis in NSCLC metastasis, we examined the levels of circPLEKHM1 and the expression of OSMR and CD206 in tumor tissues from NSCLC patients with or without metastasis (**Figure** **7**A). The results showed that the levels of circPLEKHM1, expression of OSMR, and the proportion of CD206‐positive macrophages in NSCLC patients with metastasis were markedly higher than those in NSCLC patients without metastasis (Figure 7A). Importantly, circPLEKHM1 expression was significantly and positively correlated with the proportion of CD206‐positive macrophages and OSMR expression in NSCLC patients (Figure 7B,C). NSCLC patients with high expression of circPLEKHM1, OSMR, or a high proportion of infiltrated CD206‐positive macrophages exhibited dramatically shorter overall survival and metastasis‐free survival than their control patient groups (Figure 7D–I). These results indicated the robust clinical significance of circPLEKHM1, OSMR, and M2‐type macrophages as functional biomarkers of poor prognosis in NSCLC patients. Notably, NSCLC patients with circPLEKHM1‐high, OSMR‐high, and CD206‐positive macrophages showed the worst overall survival and metastasis‐free survival (Figure 7J,K). In summary, our findings suggested that circPLEKHM1 regulates OSMR to induce macrophage polarization toward the M2 phenotype, leading to NSCLC metastasis and poor clinical outcomes in NSCLC patients. CircPLEKHM1 could serve as an important biomarker and therapeutic target in NSCLC metastasis (Figure 7L).

## 3 Discussion

Cancer metastasis is a complicated process that is closely related to intercellular communication within the hypoxic TME to mediate premetastatic niches.^[^ ^8^, ^36^ ^]^ However, the mechanisms underlying these interactions remain unclear, and strategies to block them against cancer metastasis are still lacking. Exosomes are important mediators of intercellular communication within the TME and regulate tumor progression by transferring various molecules including mRNA, miRNAs, noncoding RNAs, and proteins.^[^ ^12^, ^37^ ^]^ Particularly, some circRNAs are enriched in tumor cell‐derived exosomes,^[^ ^38^ ^]^ but little is known about their roles in regulating intercellular communication and cancer metastasis. In this study, we discovered that NSCLC‐secreted exosomal circPLEKHM1 is a hypoxia‐responsive circRNA that promotes the M2 polarization of macrophages to regulate cancer metastasis in a hypoxic TME. Exosomal circPLEKHM1 was internalized by macrophages and served as an essential component for enhancing the interaction of PABPC1 with eIF4G to promote OSMR translation, which induced the M2 polarization of macrophages to promote NSCLC metastasis. Notably, we developed a radioactive ^125^I‐CD115 probe for SPECT‐CT imaging to verify that exosomal circPLEKHM1 strongly induced M2 polarization in tumor‐associated macrophages in vivo. Importantly, the circPLEKHM1/OSMR/M2 macrophage polarization axis functioned as a significant biomarker for metastasis and poor prognosis in NSCLC patients and is an effective target to reduce NSCLC metastasis in vivo. This study revealed the detailed molecular components of the exosomal circPLEKHM1/PABPC1‐eIF4G complex involved in regulating cancer cell–macrophage crosstalk in the TME. We highlighted a new circRNA‐mediated mechanism underlying intercellular communication during hypoxia that can be effectively targeted to fight cancer metastasis.

In recent years, circRNAs have attracted increasing attention for biomedical applications because of their unique structure and stability. However, their roles in cancer cell metastasis are only beginning to be characterized.^[^ ^22^ ^]^ Addressing their physiological and pathological functions in cancer progression is critical for improving emerging attempts to develop circular RNA‐based therapeutics.^[^ ^21^ ^]^ In this study, using multiple preclinical animal models of lung cancer metastasis, we found a robust therapeutic efficacy of targeting circPLEKHM1 in inhibiting NSCLC metastasis in vivo, along with a significant reduction in M2 macrophages. Interestingly, this therapeutic efficacy was not observed upon intervention with the noncircular region of the host linear gene, PLEKHM1. Therefore, we demonstrated the potential of circPLEKHM1 as not only a functional biomarker but also an effective therapeutic target for NSCLC metastasis.

PLEKHM1 is a relatively new gene that has been identified as a multiprotein adaptor to the endolysosomal system,^[^ ^23^, ^24^, ^39^ ^]^ however, its biological functions remain largely unknown. Herein, we discovered a new function of circPLEKHM1 as a scaffold connecting the PABPC1‐eIF4G interaction for RNA regulation. OSMR is a member of the IL‐6 receptor family and has diverse cellular functions such as regulating cell growth and differentiation.^[^ ^40^ ^]^ OSMR is highly expressed in many cancer types, including ovarian, pancreatic, glioma, and breast cancer.^[^ ^41^, ^42^, ^43^, ^44^ ^]^ However, little is known about the role of OSMR in macrophages. In this study, we revealed that OSMR plays a vital role in mediating circPLEKHM1‐induced M2 macrophage polarization and JAK/STAT3 signaling pathway to promote lung cancer metastasis. Our study elucidated new molecular insights of circPLEKHM1/OSMR in macrophages, which could contribute to developing rational strategies to improve the TME.

Metastasis, particularly bone metastasis, is life‐threatening in a large proportion of NSCLC patients. There is an urgent clinical need to identify NSCLC patients who are at a high risk of metastasis and develop effective strategies to treat NSCLC metastasis. Using metastatic samples from NSCLC patients and several NSCLC metastatic mouse models, we discovered that exosomal circPLEKHM1 could serve as a valuable prognostic/predictive biomarker for NSCLC metastasis and poor survival. Moreover, circPLEKHM1‐ASO therapy markedly inhibited NSCLC metastasis, prolonged survival in vivo, and decreased OSMR‐mediated intra‐tumoral M2 macrophages. Therefore, circPLEKHM1 abundance may be a useful biomarker for predicting tumor response to potent inhibitors of M2‐type macrophage polarization, such as MEK and HDAC inhibitors,^[^ ^45^ ^]^ or future circPLEKHM1‐based drugs for the treatment of NSCLC metastasis. Based on the high stability feature of circRNAs and the sampling convenience of exosome examination from human bioliquids such as serum or plasma, future studies are needed to explore the potential utility of exosomal circPLEKHM1 extracted from human bioliquids. For instance, as a predictive and/or prognostic biomarker, it may guide treatments targeting the circPLEKHM1/OSMR/M2 macrophage polarization axis to treat lung cancer metastasis.

In conclusion, we identified exosomal circPLEKHM1 as a robust hypoxia‐induced metastasis‐promoting circRNA in NSCLC that functions as a prognostic biomarker and therapeutic target for NSCLC metastasis. Exosomal circPLEKHM1 responds to hypoxia in the TME and acts as a messenger for cancer cell‐macrophage crosstalk to promote NSCLC metastasis by enhancing the PABPC1‐eIF4G interaction as a critical scaffold to regulate OSMR‐mediated M2 macrophage polarization (Figure 7L). This study revealed a critical exosomal circRNA‐mediated mechanism in regulating intercellular communication upon hypoxia within the TME and highlighted the broad potential of circRNAs as predictive biomarkers and therapeutic targets for cancer metastasis.

## 4 Experimental Section

### Cell Lines and Cell Culture

Human monocyte/macrophage cell line THP‐1 (ATCC, Cat#TIB‐202), human non‐small cell lung cancer cell lines A549 (ATCC, Cat#CCL‐185), and H1299 (ATCC, Cat#CRL‐5803) were purchased from the American Type Culture Collection. Human lung cancer cell line PC9 (Pricella, Cat#CL‐0668) and mouse lung cancer cell line LA795 (Pricella, Cat#CL‐0376) were purchased from Pricella. Human bone mesenchymal stem cells (MSCs) were purchased from OriCell (HUXMA‐01001). Cell lines were cultured in RPMI‐1640 or F‐12K medium supplemented with 10% FBS and antibiotics, at 37 °C with 5% CO2 in normoxic (21% O2) or hypoxic (1% O2) environments. THP‐1 cells were treated with 100 ng ml^−1^ PMA for 24 h to induce macrophage differentiation.

### Co‐Culture of Stromal and NSCLC Cells

Stromal cells were initially co‐cultured in a medium containing 50 µg mL^−1^ of exosomes for 48 h using a 0.1 µM polyethylene terephthalate (PET) membrane insert (Jet Bio‐Filtration, Guangzhou, China). NSCLC cells were seeded into 24‐well plates, and then the insert containing exosome‐pretreated stromal cells was placed in a separate 24‐well plate, maintaining a tumor cell‐to‐stromal cell ratio of 1:1. Following this co‐culture setup, tests were conducted to assess the biological properties of NSCLC cells.

### Exosome Purification and Tracking Analysis

Exosomes from cells were isolated by ultracentrifugation. A549 cells were cultured in exosome depleted medium for 48 h under normoxic or hypoxic environments, and supernatants were collected for exosome purification. Briefly, supernatants were centrifuged at 300 g for 10 min to remove cells. Then supernatants were filtered through a 0.22 µm syringe filter and isolated by ultracentrifugation at 120 000 g for 90 min (4 °C). After that, exosomes were palleted by ultracentrifugation at 120 000 g (4 °C) for 90 min, followed by a wash with PBS. The isolated exosome pellets were quantified and analyzed by nanoparticle tracking analysis (NTA).

### High‐Throughput Sequencing

Next‐generation RNA sequencing assay was performed to detect the mRNA and ncRNA expression profiles at KangChen Bio‐tech (Shanghai, China) and Biomarker Technologies (Beijing, China) using Illumina HiSeq 4000 (Illumina, San Diego, CA, USA). STAR software (v2.04) was used to align the high‐quality clean reads to the human reference genome/transcriptome. Then DCC software (v0.4.4) was used to identify circRNAs and calculate the raw junction read counts. DESeq2 software (v1.28.1) was used to normalize the raw junction reads and filter the differentially expressed circRNA (fold change ≥ 2, *p*‐value ≤ 0.05).

### Flow Cytometry

THP‐1 differentiated cells induced by PMA and treated with exosomes or gene‐transfected for 48 h were washed and resuspended in cell staining buffer (1 × PBS buffer containing 1% BSA). Antibodies were added and incubated on ice for 15–20 min in the dark. Finally, the cells were washed, resuspended, and analyzed using flow cytometry (BD, USA) according to the manufacturer's instructions. The used antibodies are described in the reagents and resources file (Supporting Information).

### Wound Healing Assay

NSCLC cell migration and invasion were examined by wound healing assay and transwell assay, respectively. 4 × 10^5^ NSCLC cells were seeded into 6‐well plates and scratched at the full cell level after 24 h. Serum‐free medium was added and photographed at 0, 24, and 48 h, respectively.

### Transwell Assay

According to the manufacturer's instruction, cell migration (without Matrigel) and invasion (with Matrigel) assays were performed using Transwell 24‐well Boyden chambers (Corning, USA) with 8.0 µm pore size + polycarbonate membranes. In short, 5 × 10^4^ NSCLC cells in 200 µL of serum‐free medium were seeded in the top chamber, while 600 µL of medium with 10% FBS was added to the bottom chamber. After 48 h, cells that migrated or invaded into the bottom chamber were fixed with 4% PFA, stained with 0.1% crystal violet, and recorded by microscopy.

### Transmission Electron Microscopy (TEM)

Exosomes were fixed with 4% PFA (at 1:1 dilution) and deposited on formvar‐carbon‐coated grids at room temperature for 20 min. Next, the copper grids were fixed with 1% glutaraldehyde and washed with deionized water. Afterward, negative staining was performed with uranyl acetate for 10 min, then the excess liquid was absorbed with filter paper, and the samples were observed under a transmission electron microscope after natural drying.

### Total RNA and Exosomal RNA Extraction

Total RNA was extracted from cells or tissues using TRIzol reagent (Thermo Fisher Scientific, USA). According to the manufacturer's instructions, exosomal circRNAs were extracted from the culture medium of A549 cells culturing using an exoRNeasy Midi kit (QIAGEN, Germany). The quality and quantity of extracted RNA were evaluated by a Nanodrop 2000 Spectrophotometer (Thermo Fisher Scientific, USA).

### RNA Pull‐Down and Mass Spectrometry

Magnetic RNA‐Protein Pull‐Down Kit (Thermo Fisher, MA, USA) was used to perform circRNA pulldown. The sequences of 3′ biotin‐labeled probes (GenePharma, Shanghai, China) targeting circPLEKHM1 are ACTCTGAGATGATGTGTTCTTGCAGAGACC. The 100 nmol circRNA‐specific and NC probes were incubated with Streptavidin Magnetic Beads at room temperature for 5 h. Then, the above beads were incubated with cell lysates from circPLEKHM1‐overexpressing THP‐1 differentiated cells (3 × 10^7^) induced by PMA at 4 °C overnight. Next, the RNA binding proteins were collected with 50 µl elution buffer after being washed three times before subjecting to silver staining and mass spectrometry (Genechem, Shanghai, China).

### Reverse Transcription and RT‐qPCR

The cDNA was synthesized from mRNA and circRNAs by HiScript II Q RT SuperMix (Vazyme, Nanjing, China) under the recommended conditions. The cDNA was synthesized from mRNAs using the cDNA Synthesis Kit (Takara, Japan). Real‐time PCR was performed using a SYBR Green RT‐PCR Kit (Vazyme, Nanjing, China) on QuantStudioTM 7 Flex (Thermo Fisher Scientific). circRNAs and mRNA were normalized to β‐Actin. MiRNAs were normalized to U6. Gene expression was quantified using the 2‐∆Ct method. The sequences of specific primers or probes applied in this study are listed in the reagents and resources file (Supporting Information).

### RNase R Treatment

Total RNA extracted from cells was treated with 3 U µg^−1^ RNase R (Beypotime) for 30 min at 37 °C. Then, the relative expression levels of circPLEKHM1 and PLEKHM1 mRNA were detected by RT‐qPCR.

### Actinomycin D Assays

To verify the stability of circRNA and mRNA, cells were cultured with or without 2 µg ml^−1^ actinomycin D (Sigma‐Aldrich, MO, USA) and then the total RNA was harvested at different time points for RT‐qPCR analysis.

### Western Blotting

Cells were lysed using ice‐cold RIPA lysis buffer (Keygenbio, Nanjing, China) and the total protein concentration was quantified using a BCA assay kit (Thermo Fisher Scientific). The experiments followed the published protocol from Abcam. The used antibodies are described in the reagents and resources file (Supporting Information). Images were detected by the bioanalytical imaging system.

### Fluorescence Labeling and Intracellular Immunofluorescence

Exosomes were labeled with PKH26 red fluorescent using a staining kit (UmiBio, Cat#UR52302, Shanghai, China). According to the manufacturer's instructions, 2 µl PKH26 dye was added to 100 µg of exosomes and incubated for 5 min. Then, the THP‐1 differentiated cells induced by PMA were treated with 40 µg mL^−1^ exosomes labeled PKH26 in 12‐well plates at 37 °C for 24 h. Next, the slides were fixed with 4% formaldehyde for 20 min, and the nuclei were stained with DAPI fluorescent dye (Sigma‐Aldrich, Cat#D9542, USA). Intracellular immunofluorescence was used to examine the expression of PABPC1 and eIF4G followed by staining with DAPI. All images were taken by the Olympus FluoView FV1000 confocal microscope (Olympus, London, England).

### Fluorescent In Situ Hybridization (FISH)

The sections were subjected to hybridization with Cy3‐labeled probes (RiboBio) that targeted the junction site of circPLEKHM1, using a FISH Kit (RiboBio, Cat#C10910) in accordance with the manufacturer's instructions. The sections were incubated with PABPC1‐specific antibodies at 4 °C overnight, followed by FITC‐conjugated secondary antibodies.

### RIP

RIP was a technique used to identify RNA molecules that interacted with specific RNA‐binding proteins (RBPs). In this study, the Magna RNA‐Binding Protein Immunoprecipitation Kit (Sigma) was used to perform RIP. The cells were lysed in a complete RIPA buffer containing a protease inhibitor cocktail and RNase inhibitor. The cell lysates were then incubated with RIP buffer containing magnetic beads conjugated with antibodies. The samples were digested with proteinase K to isolate the immunoprecipitated RNA. The purified RNA was then subjected to real‐time PCR to demonstrate the presence of the binding targets. The used antibodies are described in the reagents and resources file (Supporting Information).

### Chromatin Immunoprecipitation (ChIP) Assay

The ChIP experiment was conducted using a ChIP assay kit (Beyotime, Cat#P2078) in accordance with the manufacturer's instructions. A549 cells were initially cross‐linked with formaldehyde and sonicated to achieve an average size of 200‐to‐500 base pairs. Prior to incubation with protein A/G beads coated with the anti‐HIF1A antibody (Abcam, Cat#ab51608), cell lysates were precleared with protein A/G beads. Anti‐rabbit IgG was utilized as a negative control. The DNA released from the protein‐DNA complex was cross‐linked and purified using a DNA Extraction Kit (GeneMark), and the eluted DNA was subsequently subjected to RT‐qPCR.

### Dual‐Luciferase Reporter Assay System

A promoter region of PLEKHM1 was constructed into pGL3‐based vectors, containing putative binding areas for HIF1A, namely the wild type (WT) and mutant type (MUT). To investigate the impact of HIF1A on the PLEKHM1 promoter, pGL3‐based constructs containing either the WT or MUT promoter sequences, along with a Renilla luciferase reporter plasmid, were individually transfected into HEK293T cells. After 48 h of culture under normoxia or hypoxia, Firefly and Renilla luciferase activity were measured using a dual‐luciferase reporter assay system (Promega, Cat#E1910). The ratio of Firefly luciferase to Renilla activity was calculated for each sample.

### Polysome Profiling

Cells at ≈80% confluence were pretreated with 100 µg mL^−1^ of cycloheximide (CHX) at 37 °C for 5 min. Following this, the cells were harvested and incubated in 800 µL of polysome cell extraction buffer, which contained 10 mM NaCl, 10 mM MgCl2, 10 mM Tris–HCl (pH 7.5), 1% Triton X‐100, 1% sodium deoxycholate, 0.2 U µL^−1^ RNase inhibitor, 1 mM DTT, and 0.1 mg mL^−1^ cycloheximide. After a centrifugation step at 16 000 × g for 10 min at 4 °C, the resulting supernatants were collected and layered onto 10–50% sucrose gradients. Ultracentrifugation was then carried out at 274 000 g using a Beckman SW41 rotor for 1 h and 40 min at 4 °C. The samples were fractionated using a density gradient fractionation system provided by Biocomp, Canada, with absorbance monitored at 254 nm. Finally, the collected fractions were subjected to RT‐qPCR analysis.

### Clinical Samples

Specimens were obtained from 110 patients who underwent surgery between January 2010 and July 2015, from the surgical specimen archives of Fudan University Shanghai Cancer Center. Patients' follow‐up visits continued until June 2020, with data being censored at the last follow‐up visit or at the time of the patient's death without relapse. No pre‐operative treatment was administered to the recruited patients. This study was approved by the institutional clinical research ethics committees of Fudan University Shanghai Cancer Center (050432‐4‐2108*). Written informed consent was obtained from each patient, and the study was conducted in accordance with the International Ethical Guidelines for Biomedical Research Involving Human Subjects (CIOMS).

### Animal Experiments

To conduct the experimental metastasis assay, cancer cells in 100 µl of PBS were injected into the left cardiac ventricle of BALB/c nude mice (7–8 weeks old). For intratibial implantation of cancer cells, cells were injected into the tibia at a final concentration of 100 000 cells per 10 µl. To ensure successful injection, photon flux from the whole body of the mice was immediately monitored after injection. The growth of tumors in the bones was monitored and quantified by measuring luminescence using in vivo bioluminescence imaging with the IVIS Spectrum Imaging System (PerkinElmer). For the in vivo circPLEKHM1‐ASO treatment assay, the mice were treated with circPLEKHM1‐ASO by injecting them into the tibias or tail vein every 2 days. After 3 weeks, the bones were extracted and sectioned with a diamond knife. The sections were stained for HE and TRAP. All in vivo experiments were carried out in accordance with the requirements of the Animal Research Committee of Fudan University on the care and use of experimental animals in research (FUSCC‐IACUC‐S2022‐0565).

### Micro‐CT Scan

The bone of the mouse was scanned by micro‐CT in vivo (1272 Skyscan, Bruker) with a 0.25 µm aluminum filter at 60 kV, using a detection pixel size of 9 µm. The reconstructed images were generated using Skyscan Recon software following the manufacturer's guidelines and were analyzed using DataViewer software (Bruker) and CTVox software (Bruker).

### Synthesis and Stability Assessment of ^125^I‐CD115 for SPECT/CT Scan

The catalyst iodogen was used to label CD115 with radioactive isotope ^125^I, resulting in the synthesis of ^125^I‐labeled CD115. The procedure involved adding an aqueous solution of CD115 to an Eppendorf tube containing iodogen, followed by the addition of a solution of free ^125^I. The solution was allowed to stand at room temperature for 30 min for radiosynthesis. The in vitro stability of ^125^I‐CD115 was assessed by co‐culturing it with fetal bovine serum (FBS) or phosphate‐buffered saline (PBS) for 2, 4, 6, 12, 24, 48, and 72 h. The labeling yield and stability were determined by instant thin‐layer chromatography.

### Biodistribution of ^125^I‐CD115

Following inoculation with lung cancer cells, ^125^I‐CD115 (^125^I: 0.69 MBq; CD115: 80 µg) was administered to a NSCLC tumor‐bearing mouse via the caudal vein for biodistribution analysis. Tumors and major organs of the mouse were collected, weighted, and counted using a gamma counter at designated time intervals (6, 24, 48, and 72 h). The results were reported as %ID g^−1^ of tissue.

### Micro‐SPECT/CT Scan

Micro‐SPECT/CT imaging was performed on an NSCLC tumor‐bearing mouse using a specialized small animal scanner (nanoScan@SC SPECT/CT 4 Detector; Mediso Medical Imaging Systems, Budapest, Hungary). To minimize the uptake of free iodine by thyroid tissue, the mouse was administered with potassium iodide (KI) solution (0.5 g mL^−1^) in drinking water 7 days prior to imaging. Following the injection of ^125^I‐CD115 via the caudal vein in tumor‐bearing mice, systemic micro‐SPECT/CT images were captured. Each CT scan (50 kV, 980 µA) lasted for 6 min, followed by a SPECT/CT acquisition of 20–40 min (matrix: 128 × 128, frame time: 30–60 s). The TeraTomo 3D (TT3D) dynamic range was used to reconstruct SPECT/CT data, which were then analyzed using Nucline 3.00 (Mediso Medical Imaging Systems). Regions of interest were plotted, with contralateral front‐limb muscles selected as the background for SPECT/CT images. Results were expressed as target‐to‐normal tissue ratios (TNR).

### Statistical Analysis

Statistical analysis was performed using GraphPad Prism 8.0.4 software (GraphPad Inc., La Jolla, CA, USA). Data were obtained from at least three independent experiments and presented as mean ± SD. Statistical analysis for two‐group comparisons was conducted using the two‐tailed Student's *t*‐test or non‐parametric Mann–Whitney test. One‐way ANOVA was used to compare data from more than two groups under a single treatment, whereas two‐way ANOVA was employed to compare data from more than two groups under multiple treatments. Survival curves were generated using the Kaplan–Meier method and analyzed using the log‐rank test. Correlation analysis was performed using Pearson's correlation coefficient. A *p*‐value of less than 0.05 was considered statistically significant, denoted as *p < 0.05, **p < 0.01, and ***p<0.001.

## Conflict of Interest

The authors declare no conflict of interest.

## Author Contributions

D.W. and S.W. contributed equally to this work. S.T. and S.S. designed and led the project. D.W., S.W., M.J., J.W., X.L., X.T., W.T., and Y.Z. performed experiments. D.W., S.W., M.J., Y.N., Q.Z., J.C., and Y.Z. analyzed data. S.W. and W.Y. provided clinical samples and information. S.T., S.S., G.H., and H.W. guided data analysis. D.W., S.T., and H.Y. wrote the manuscript. All authors contributed to data interpretation and discussion of results on the manuscript.

## Supporting information

Supporting Information

Supporting Information

## Data Availability

The data that support the findings of this study are available in the Supporting Information.
