# Supplementary material for: Hypoxic Exosomal circPLEKHM1‐Mediated Crosstalk between Tumor Cells and Macrophages Drives Lung Cancer Metastasis
Source: Adv Sci (Weinh). 2024 Mar 21;11(22):2309857. doi: 10.1002/advs.202309857 (PMC11165461; doi:10.1002/advs.202309857)
Supplement: Supplementary file 1 — Supporting Information [file ADVS-11-2309857-s001.pdf]

ADVANCED SCIENCE
Open Access

Supporting Information

for *Adv. Sci.*, DOI 10.1002/advs.202309857

Hypoxic Exosomal circPLEKHM1-Mediated Crosstalk between Tumor Cells and Macrophages Drives Lung Cancer Metastasis

Dongliang Wang, Shuoer Wang, Mingming Jin, Yan Zuo, Jianpeng Wang, Ya Niu, Qian Zhou, Jiwei Chen, Xinru Tang, Wenxuan Tang, Xiyu Liu, Hang Yu, Wangjun Yan, Huan-Huan Wei, Gang Huang, Shaoli Song and Shuang Tang**

Hypoxic exosomal circPLEKHM1-mediated crosstalk between tumor cells and macrophages drives lung cancer metastasis

Supplementary methods

Cell isolation

PBMCs (Peripheral Blood Mononuclear Cells) were obtained from healthy donors using Ficoll (PromoCell, Heidelberg, Germany) density gradient centrifugation. To positively select CD14+ monocytes, anti-CD14 magnetic beads (Miltenyi Biotec, Auburn, CA) were used following the manufacturer’s instructions. A total of 105 CD14+ monocytes were seeded per well (24-well dish) on FBS-coated surfaces in RPMI-1640/10% fetal bovine serum (FBS) supplemented with 50 ng/ml MCSF (PeproTech) for a period of 7 days to facilitate their differentiation into M0 macrophages.

The primary tumors from tumor-bearing mice were collected and processed using previously established procedures to isolate tumor infiltrated macrophages. In brief, the tumors were mechanically dissociated and subjected to digestion with collagenase type IV (1 mg/mL) and DNase I (2 U/mL) at 37°C for 60 minutes. The resulting cell suspension was then filtered using a 40 μm cell strainer. For the isolation of macrophages, the cells were stained with antibodies and sorted via flow cytometry into the following subtypes: M1 macrophages (F4/80+CD11b+CD86+) and M2 macrophages (F4/80+CD11b+CD206+).

CAFs (Cancer-Associated Fibroblasts) were isolated from tumor samples obtained from patients. The tumor tissues were subjected to an 8-hour digestion with 1 mg/ml collagenase I (YEASEN Biotech, Shanghai, China). Subsequently, the

digested CAFs were filtered through a 200-mesh filter into a culture medium composed of DMEM (Dulbecco's Modified Eagle Medium) supplemented with 15% FBS.

HUVECs (Human Umbilical Vein Endothelial Cells) were purchased from OriCell (HUVEC-20001).

Supplementary figures and figure legends

Figure S1

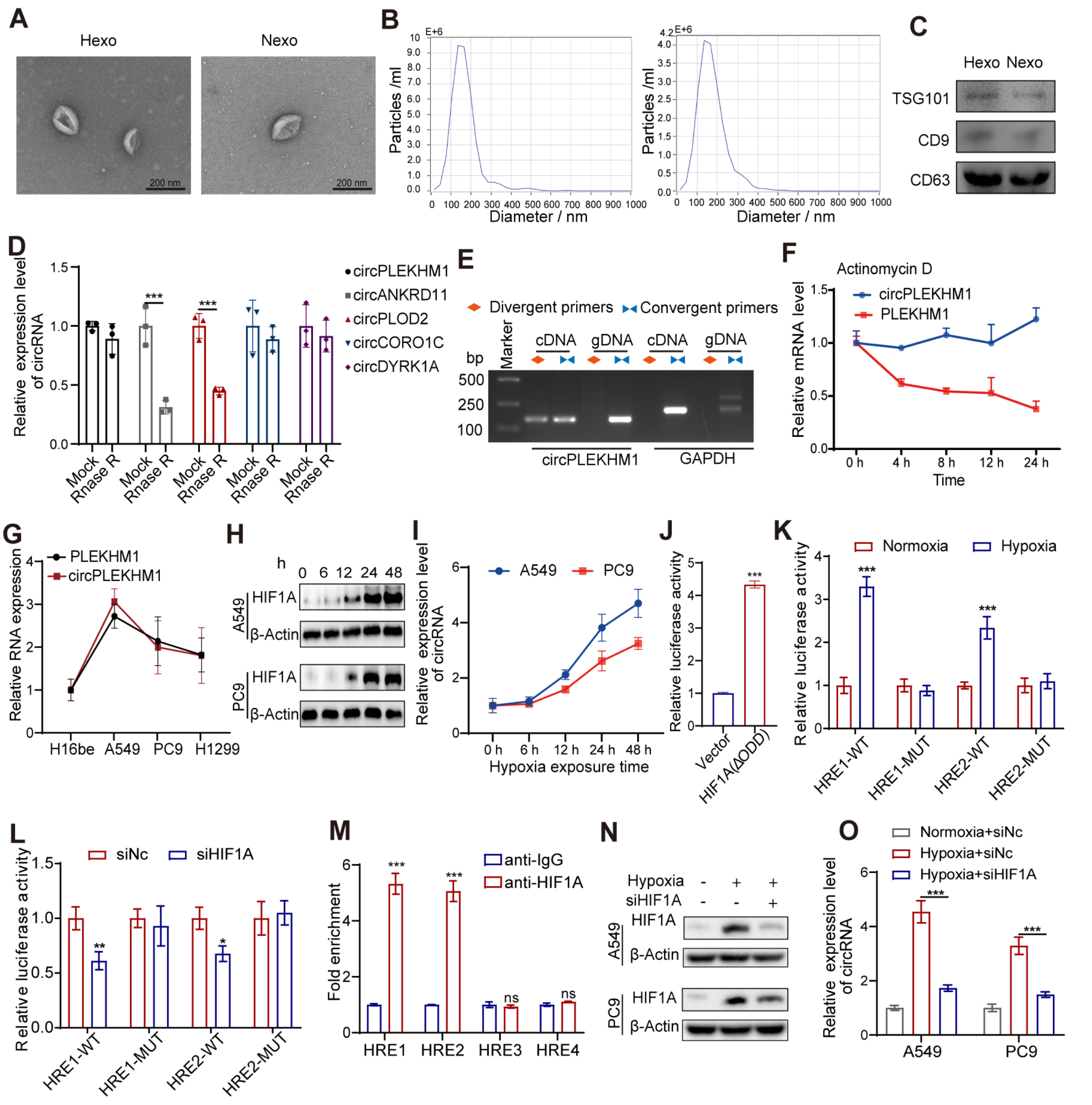

Figure S1. CircPLEKHM1 is enriched in hypoxic NSCLC-derived exosomes and characteristic of circPLEKHM1

A-C) Identification of exosomes derived from normoxic and hypoxic A549 cells by TEM (A), NTA (B) and western blot (C).

D) CircRNAs expression levels in hypoxic A549 cells treated with Rnase R, measured by RT-qPCR.

E) Divergent primers for circPLEKHM1 could be amplified from cDNA but not gDNA using PCR analysis. The divergent primers for GAPDH could not be amplified.

F) After treated with actinomycin D, circPLEKHM1 was more stable than linear PLEKHM1 transcripts. The indicated RNA was extracted from cells at different times for RT-qPCR.

G) CircPLEKHM1 and PLELKHM1 expression levels in H16be, A549, PC9 and H1299 cells verified by RT-qPCR.

H) The HIF1A protein level in NSCLC cells exposed to hypoxia for different time.

I) CircPLEKHM1 expression level in NSCLC cells exposed to hypoxia for indicated time.

J) Luciferase intension of 293T cells co-transfected with luciferase reporter plasmids and HIF1A (ΔODD).

K) Luciferase intension of 293T cells co-transfected with indicated luciferase reporter plasmids under hypoxia or normoxia.

L) Luciferase intension of 293T cells co-transfected with indicated luciferase reporter plasmids upon knockdown of HIF1A.

M) ChIP assays to evaluate the interactions between HIF1A and HREs in PLEKHM1 promoter region.

N) The HIF1A protein level in NSCLC cells transfected with siHIF1A and exposed to hypoxia.

O) Abundance of hypoxia-induced circPLEKHM1 was inhibited by knockdown of HIF1A.

Data in D, J-M were presented as mean ± SD, *p < 0.05, **p < 0.01, ***p < 0.001 by two-tailed student's t-test. Data in O were presented as mean ± SD, ***p < 0.001 by one-way ANOVA test.

Figure S2

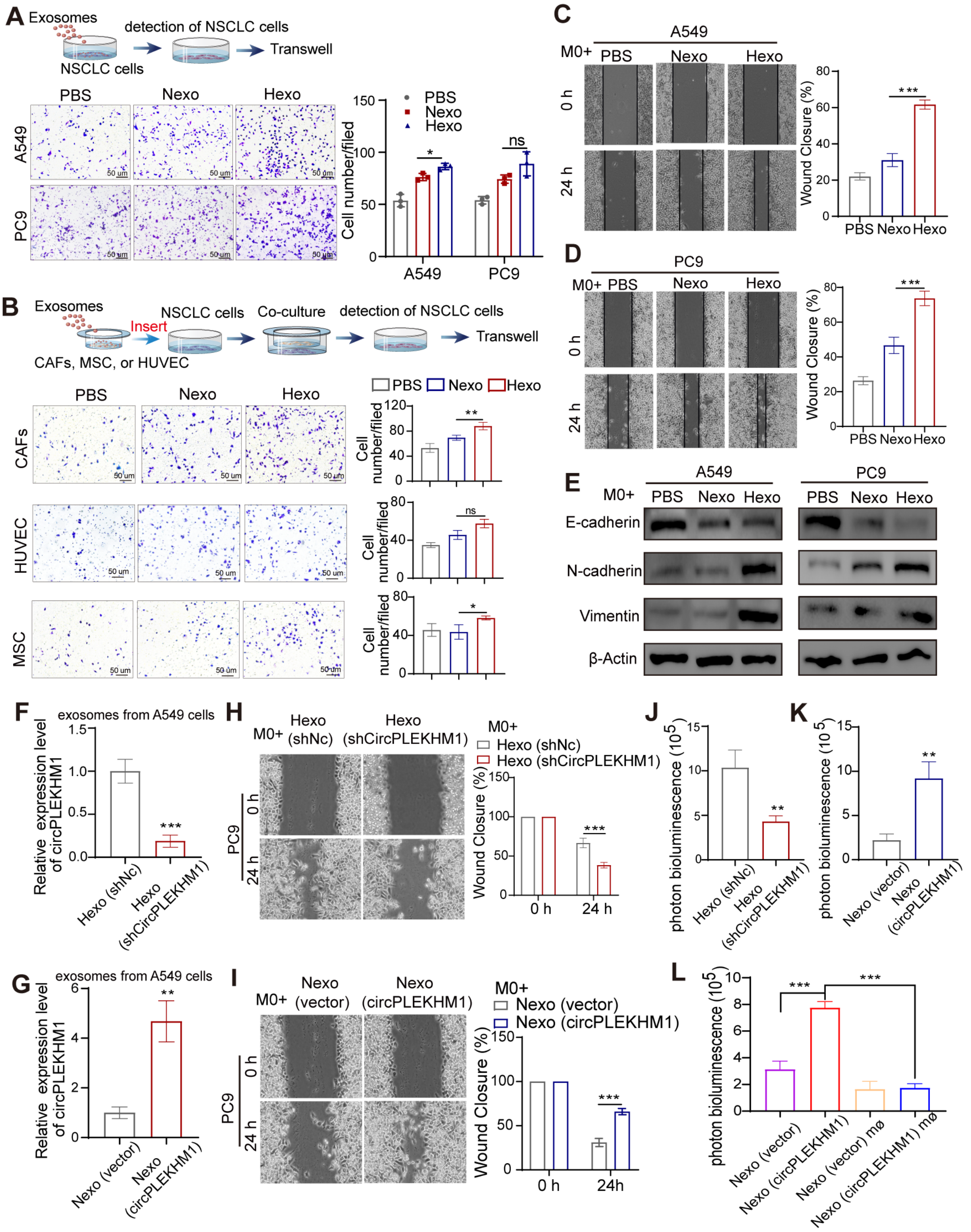

Figure S2. Hypoxic exosomal circPIEKHM1 promotes NSCLC metastasis through macrophages

A) Illustration of exosomes impacting on NSCLC cells (upper). Transwell migration assay of A549 and PC9 cells treated with indicated exosomes (lower). Scale bar, 50μm.

B) Schematic illustration of indirect co-culture experiment. CAFs, HUVEC or MSC were pre-treated with Nexo or Hexo exosomes and then co-cultured with A549 cells, following with transwell assay to detect A549 cell migration (upper). Transwell migration results of A549 cells co-cultured with CAFs, HUVEC or MSC treated with indicated exosomes (lower). Scale bar, 50μm.

C,D) Wound healing assay of A549 and PC9 cells co-cultured with M0 macrophages that was pre-treated with indicated exosomes.

E) A549 and PC9 cells were co-cultured with M0 macrophages pre-treated with indicated exosomes, and the expression of metastasis-related proteins was detected by Western blotting.

F) Back-splicing sequence-targeting shRNAs effectively knockdown circPLEKHM1 in exosomes derived from hypoxic A549 cells, verified by RT-qPCR.

G) Overexpression of circPLEKHM1 to upregulate circPLEKHM1 in exosomes derived from normoxic A549 cells, verified by RT-qPCR.

H-I) Wound healing assay of the constructed PC9 cells as indicated co-cultured with M0 macrophages treated with indicated exosomes.

J-L) Quantifications of the luciferase activity by IVIS Bioimager on mice bearing tumors (n=5).

Data in A-D, L were presented as mean ± SD, *p < 0.05, **p < 0.01, ***p < 0.001 by one-way ANOVA test. Data in F-I were presented as mean ± SD, *p < 0.05, **p < 0.01, ***p < 0.001 by two-tailed student's t-test. Data in J-K were presented as mean ± SD, **p < 0.01 by Mann-Whitney test.

Figure S3

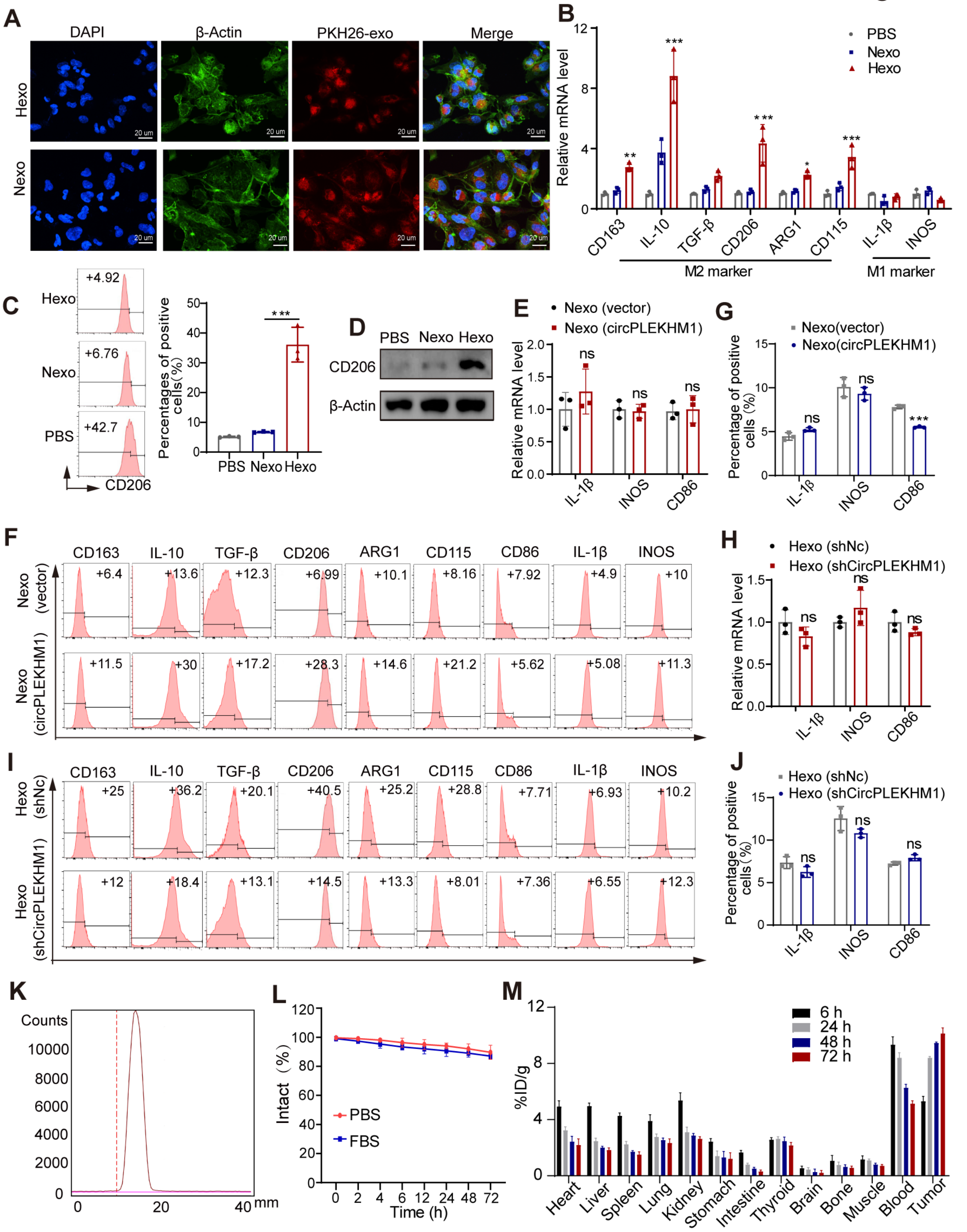

Figure S3. Exosomal circPIEKHM1 induces macrophage polarization towards to M2-type.

A) Fluorescence images of M0 cells treated with PKH26-stained Nexo and Hexo (red), and then stained with phalloidin (green) and DAPI (blue) for visualization of exosomes uptake. Scale bar, 20 μm.

B) RT-qPCR assay for *CD163*, *IL-10*, *TGF-β*, *CD206*, *ARG1*, *CD115*, *IL-1β* and *INOS* mRNA levels in cells treated with the indicated exosomes.

C) Flow cytometry was performed to analyze the effect of indicated exosomes on the expression of the typical M2 marker CD206 in macrophages.

D) Western blot analysis to detect the expression of CD206 in macrophages treated with indicated exosomes.

E-J) RT-qPCR assay and Flow cytometry staining for CD163, IL-10, TGF-β, CD206, ARG1, CD115, CD86, IL-1β and INOS to analyze the effect of indicated exosomes on macrophages.

K) Initial labeling yield of ^{125}I-CD115 placed for 30 min at room temperature.

L) Radiolabeling stability of ^{125}I-CD115 co-incubated in 10% FBS or PBS over time.

M) Biodistribution of ^{125}I-CD115 in mice bearing tumors at 6, 24, 48, and 72 h post-injection.

Data in B-C were presented as mean ± SD, *$p < 0.05$, **$p < 0.01$, ***$p < 0.001$ by one-way ANOVA test. Data in E, G, H, J were presented as mean ± SD, ***$p < 0.001$ by two-tailed student's t-test.

Figure S4

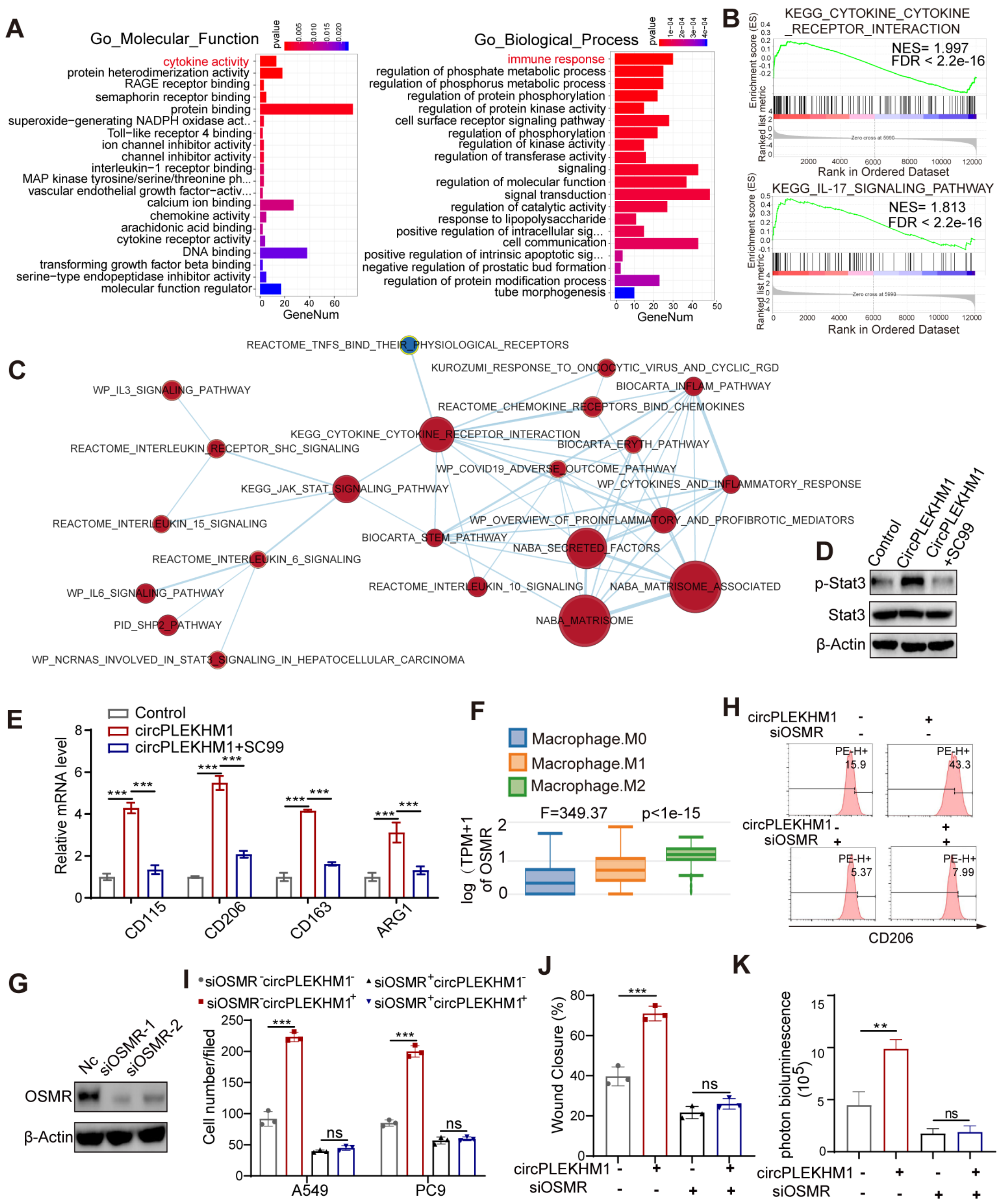

Figure S4. CircPIEKHM1 promotes macrophages M2 polarization by OSMR/JAK/STAT axis

A) GO (Gene Ontology) analysis of DEGs.

B) GSEA (Gene Set Enrichment Analysis) for Cytokine-cytokine receptor interaction and IL-17 signaling pathway of DEGs.

C) GSEA similarity network analysis of upregulated (red) and downregulated (blue) pathways centered on JAK-STAT pathway.

D) Western blot analysis to detect protein expression of p-Stat3 in macrophages transfected with circPLEKHM1 or treated with SC99.

E) RT-qPCR assay for CD115, CD206, CD163 and ARG1 mRNA levels in treated cells.

F) Expression of OSMR in M0, M1 and M2 macrophages using GEPIA database.

G) Immunoblot analysis for OSMR in cells transfected with siOSMR.

H) Flow cytometry to detect CD206 in macrophages under the indicated intervention.

I) Quantification of transwell migration assay of A549 and PC9 cells co-cultured with IL4-treated macrophages transfected with circPLEKHM1 and/or siOSMR.

J) Quantification of wound healing assay of A549 cells co-cultured with IL4-treated macrophages transfected with circPLEKHM1 and/or siOSMR.

K) Quantifications of the luciferase activity by IVIS Bioimager on mice bearing tumor co-cultured with macrophages under the indicated intervention (n=5).

Data in E, I, J-K were presented as mean ± SD, $^{**}p < 0.01$, $^{***}p < 0.001$ by one-way ANOVA test.

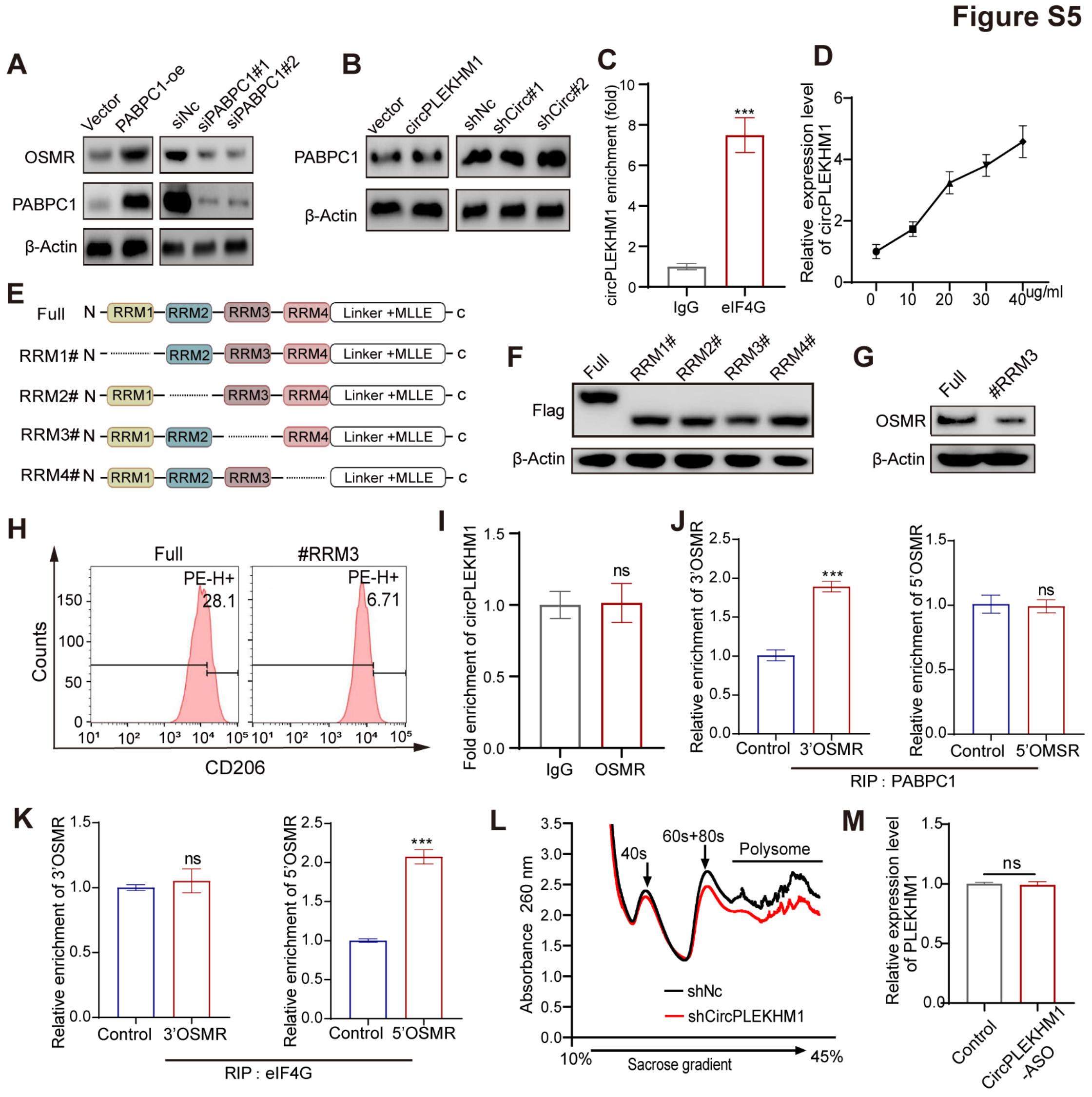

Figure S5. CircPLEKHM1/PABPC1/eIF4G RNA-protein ternary complex stabilizes OSMR mRNA and promotes its expression

A) Western blot for OSMR and PABPC1 in macrophages transfected with PABPC1 or siPABPC1.

B) Immunoblotting for PABPC1 in macrophages transfected with cirPLEKHM1 or sh-cirPLEKHM1.

C) RT-qPCR analysis of circPLEKHM1 derived from RNA immunoprecipitation (RIP)

assay with anti-eIF4G antibody.

D) CircPLEKHM1 expression levels in macrophages treated with indicated dose of hypoxic exosomes, verified by RT-qPCR.

E) Schematic for PABPC1-domain-deletion mutants.

F) Western blot analysis for Flag in cells transfected with Flag-tagged PABPC1 domain-deletion mutants.

G-H) Compared with wild-type PABPC1, RRM3-mutant PABPC1 reduced OSMR expression as detected by western blot (G), and expression of CD206 as measured by flow cytometry (H).

I) RT-qPCR analysis of circPLEKHM1 derived from RNA immunoprecipitation (RIP) assay with anti-OSMR antibody.

J) RNA immunoprecipitation (RIP) was conducted using a PABPC1 antibody in cells transfected with 3'OSMR and 5'OSMR fragments, followed by RT-qPCR analysis of OSMR mRNA.

K) RNA immunoprecipitation (RIP) was conducted using a eIF4G antibody in cells transfected with 3'OSMR and 5'OSMR fragments, followed by RT-qPCR analysis of OSMR mRNA.

L) Polysome profiling of cells with circPLEKHM1 knockdown of OSMR mRNA distribution in different ribosome fractions.

M) RT-qPCR analysis of PLEKHM1 in circPLEKHM1-ASO treated cells.

Data in C, I-K, M were presented as mean ± SD, ***$p < 0.001$ by two-tailed student's t-test.
