# Supplementary material for: Hypoxic Exosomal circPLEKHM1‐Mediated Crosstalk between Tumor Cells and Macrophages Drives Lung Cancer Metastasis
Source: Adv Sci (Weinh). 2024 Mar 21;11(22):2309857. doi: 10.1002/advs.202309857 (PMC11165461; doi:10.1002/advs.202309857)
Supplement: Supplementary file 2 — Supporting Information [file ADVS-11-2309857-s002.pdf]

ADVANCED SCIENCE
Open Access

Supporting Information

for *Adv. Sci.*, DOI 10.1002/advs.202309857

Hypoxic Exosomal circPLEKHM1-Mediated Crosstalk between Tumor Cells and Macrophages Drives Lung Cancer Metastasis

Dongliang Wang, Shuoer Wang, Mingming Jin, Yan Zuo, Jianpeng Wang, Ya Niu, Qian Zhou, Jiwei Chen, Xinru Tang, Wenxuan Tang, Xiyu Liu, Hang Yu, Wangjun Yan, Huan-Huan Wei, Gang Huang, Shaoli Song and Shuang Tang**

Table S1. Logistical analysis of clinicopathological features associated with metastasis of NSCLC patients.

Variables	Univariate analysis P value	OR (95% CI)	Multivariate analysis P value	OR (95% CI)
CircPLEKHM1 (high vs. low)	0.008	2.868 (1.311-6.272)	0.031	2.486 (1.089-5.679)
Age (≥55 vs. <55)	0.174	0.585 (0.270-1.268)	0.111	0.506 (0.219-1.168)
Sex (female vs. male)	0.492	1.304 (0.611-2.783)	0.794	1.116 (0.491-2.536)
T stage (III–IV vs. I–II)	0.004	3.148 (1.430-6.931)	0.014	2.841 (1.240-6.509)
